# Observation of interaction-induced modulations of a quantum Hall liquid's area

**DOI:** 10.1038/ncomms12184

**Published:** 2016-07-11

**Authors:** I. Sivan, H. K. Choi, Jinhong Park, A. Rosenblatt, Yuval Gefen, D. Mahalu, V. Umansky

**Affiliations:** 1 Braun Center for Submicron Research, Department of Condensed Matter Physics, Weizmann Institute of Science, Rehovot 76100, Israel

## Abstract

Studies of electronic interferometers, based on edge-channel transport in the quantum Hall effect regime, have been stimulated by the search for evidence of abelian and non-abelian anyonic statistics of fractional charges. In particular, the electronic Fabry–Pérot interferometer has been found to be Coulomb dominated, thus masking coherent Aharonov–Bohm interference patterns: the flux trapped within the interferometer remains unchanged as the applied magnetic field is varied, barring unobservable modulations of the interference area. Here we report on conductance measurements indicative of the interferometer's area ‘breathing' with the variation of the magnetic field, associated with observable (a fraction of a flux quantum) variations of the trapped flux. This is the result of partial (controlled) screening of Coulomb interactions. Our results introduce a novel experimental tool for probing anyonic statistics.

## Abstract (web summary)

Quantum Hall liquids play host to a wide range of unusual physics. Here, the authors use an electronic Fabry-Pérot interferometer to observe modulations of a quantum Hall liquid's area, which can offer a means to study the statistics of fractional charges.

## Graphical abstract

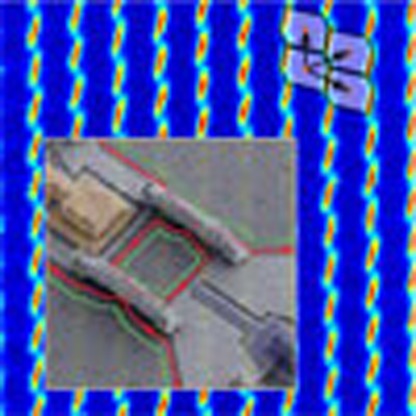

The behaviour of electrons and quasi-particles in mesoscopic systems stems from the combination of their wave-nature, particle-nature and the effect of Coulomb interactions. Electronic Fabry–Pérot interferometers (FPIs), and more generally ring-like geometries, have been utilized for the investigation of all these three facets^1^,^2^,^3^,^4^ and the rich interplay among them^5^,^6^,^7^,^8^,^9^,^10^. In particular, electronic FPIs have possibly been the most studied candidates for probing belian and non-abelian anyonic statistics of quasi-particles in the fractional quantum Hall effect (FQHE) regime^11^,^12^,^13^,^14^. Notwithstanding considerable efforts, the experimental study of anyonic statistics is still lagging behind theory, mostly owing to the adverse effect of Coulomb interactions. Two distinct regimes in the operation of FPIs—characterized by the conductance oscillation frequencies with the applied flux—have been reported^7^,^8^ and studied theoretically^15^,^16^,^17^,^18^,^19^. In the coherent AB regime, one period of these oscillations corresponds to the increase of the threaded flux through the interferometer's area by a single flux quantum *φ*_0_=*h*/*e* (*e*—electron charge and *h*—Planck's constant)^20^. On the other hand, in the Coulomb-dominated (CD) regime, it is the mutual capacitance between the device and a modulation gate that dictates the oscillation periodicities. Interference in the AB regime is harder to observe^7^,^8^, and has not been achieved thus far in any fractional quantum Hall state. At the same time, the CD regime has its own disadvantage—it does not and cannot reveal the anyonic statistics.

The AB phase underlying interference patterns evolves as 2*πδφ*_AB_/*φ*_0_=2*π*(*AδB*+*BδA*_0_)/*φ*_0_, where *δφ*_AB_ is the variation of the threaded flux through the FPI, *B* the applied magnetic field, *A* the area enclosed by the interfering edge channel and *δA*_0_=*α̇δV*_MG_ is the area variations due to the modulation gate (but excluding the effect of Coulomb interaction as explained below). Here *α* is the edge-channel modulation-gate mutual capacitance. The AB oscillations with *δB* and *δV*_MG_ are hence characterized by the following frequencies:


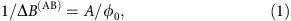



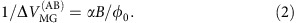


By contrast, in the CD regime with the outer most edge channel interfering, there is no *B* dependence of the conductance,


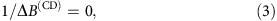



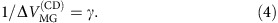


Here, 1/*γ* is the voltage required to remove a single electron from the FPI^3^,^4^,^5^,^6^, which does not depend upon the magnetic field (in similitude to the single-electron transistor^21^).

Seemingly, these two regimes may seem to be of very different nature: while the AB oscillations are due to coherent interference, the CD oscillations reflect solely the electron occupancy of the device and do not contain any information regarding the phase acquired by the interfering particles. This interpretation turned out to be too naive. A unified theoretical framework has suggested that the CD oscillations may, in fact, be AB oscillations modified by Coulomb interaction^15^. According to this interpretation^7^,^8^,^15^, as the magnetic field increases (decreases) so that the AB phase varies by *AδB*, the area will shrink (inflate) by *δA*_int_ to keep a constant flux *AδB*+*BδA*_int_=0, where the subscript ‘int' stands for ‘interactions'. This variation of the area guarantees that the charge enclosed in the interfering area is kept constant, minimizing the charging energy of the device. Then, once the flux variation reaches the value of a whole flux quantum, *AδB*=*φ*_0_, an electron abruptly ‘jumps' from the bulk into the edge and the original area is restored^7^,^15^.

According to this description, the area enclosed by the outer edge channel is ‘breathing'; on increasing the magnetic field it continuously shrinks and then abruptly inflates, altogether with periodicity Δ*B*=*φ*_0_/*A*. Nonetheless, the breathing in the CD regime has never been experimentally confirmed. The reason is that the measured conductance is simply constant, as the magnetic field is varied. Basically, the area response to increasing the magnetic field fully compensates the effect of the latter. The subsequent abrupt addition of an electron leads to a 2*π* jump in the interference phase—clearly an unobservable effect as well. We are thus ‘blind' to this phenomenon of area breathing.

Here we report on the realization of a novel device that combines the advantages of AB interferometers with CD response. It operates in an intermediate regime—in which the area response does not fully compensate the variations of the magnetic field, rendering the area ‘breathing' observable. In other words, 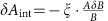, with 0<*ξ*<1, leading to a total phase evolution 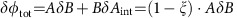. Our main findings concerning this ‘breathing' are summarized in equations (8, 9, 10) below. Measurements were performed on three different devices showing three distinct behaviours: AB dominated, namely, *ξ*=0 with no area breathing; CD, namely, *ξ*=1 with invisible breathing of the area; and an intermediate one (*ξ*=0.75), with clear area breathing. We present conductance measurements of this novel device that incorporates both AB and CD frequencies (equations (1, 2, 3, 4)), hence contain information regarding both interference and interactions, and the interplay between them. Moreover, employing detailed analysis based on the system's energy, we deduce the device's charge stability diagram, providing an insight to its rich physics being ‘hidden' in the previously reported AB and CD devices. Furthermore, we provide proposals for utilizing our device for probing anyonic statistics of quasi-particles in the FQHE regime.

## Results

### Experimental set-up

**Figure 1:**
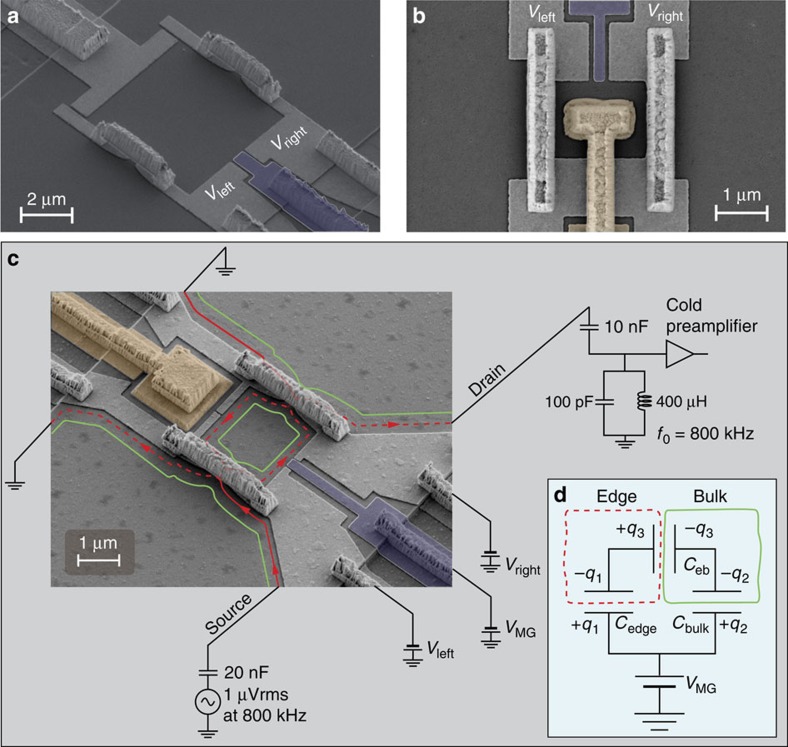
Scanning electron microscopy images and an illustration of the experimental set-up. (**a**) Bare FPI; such devices show distinct Coulomb dominated (CD) behaviour. (**b**) FPI with a grounded Ohmic contact in its centre (gold, false colour) aimed at screening Coulomb interactions; such devices show Aharonov–Bohm (AB) interference. (**c**) FPI with a grounded Ohmic contact in its close proximity (gold, false colour), inducing partial screening of Coulomb interactions; such devices show intermediate behaviour between the CD and AB. Red lines represent edge states and arrows represent the current's chirality. Current is injected into the device from the source, partitioned at the two QPCs, and probed at the drain employing a cold amplifier. Partitioned current is denoted by dashed lines. Modulation gates are marked by blue (false colour) in all images. (**d**) An equivalent electric circuit model describing the interferometer system with filling factor 1<*v*_B_<2. The system consists of the lowest Landau level's edge (denoted as ‘edge'), the bulk (denoted as ‘bulk') and the modulation gate. The mutual capacitances between all three, *C*_eb_, *C*_edge_ and *C*_bulk_, are marked on top of the circuit. The excess charge on the edge is denoted *δQ*_edge_=*δq*_3_−*δq*_1_, while the excess charge in the bulk is 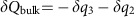. Further details on the capacitive model are provided in analysis, as well as in Supplementary Note 2 and Supplementary Fig. 5.

The devices were fabricated on a GaAs-AlGaAs heterostructure, embedding a two-dimensional (2D) electron gas with electron density ∼2 × 10^11^ cm^−2^. Electron-beam and optical lithography were employed in the fabrication process, and measurements were performed at electron temperature ∼30 mK. All FPIs consist of a pair of quantum point contacts (QPCs), playing the role of semi-transparent mirrors (Fig. 1). Three different types of devices are shown in Fig. 1. In Fig. 1a, we show the simplest realization of a FPI; such FPIs have high charging energy, regardless of their size^7^,^8^,^22^, thus showing CD behaviour. Tto suppress Coulomb interactions, a small Ohmic contact (106 nm Au/53 nm Ge/40 nm Ni with surface area 0.5 μm^2^ alloyed to the heterostructure) is placed at the centre of the interferometer's bulk^22^ (Fig. 1b, gold, false colour; see Methods for more information regarding the Ohmic contact). Such devices show AB behaviour^22^. A third type of device, not reported previously (to the best of our knowledge), consists of placing such Ohmic contacts in the vicinity of the FPI, as depicted in Fig. 1c. Unexpectedly, we have found that in such a device, the suppression of the Coulomb interactions is less efficient than the centre Ohmic contact, yet it is still significant, giving rise to a new regime intermediate between AB and CD. The role of this Ohmic contact placed in the device's vicinity is to screen the Coulomb interactions via its capacitance to the interferometer's bulk (increasing *C*_bulk_ while maintain *C*_eb_ intact, see definitions in Discussion below and more details in Methods), effectively lowering the device's charging energy and decreasing the interaction parameter *ξ*. All three devices were fabricated on the same heterostructure and designed to have the same size *A*≈2.5 μm^2^ so that they differ merely by the presence and the position of the Ohmic contacts. A fourth type of device, with the Ohmic contact inside its bulk, and surrounded by an additional gate was found to operate in the intermediate regime as well, and is discussed in Supplementary Note 1 and Supplementary Figs 1–3. All measurements were conducted at bulk fillings 1<*ν*_B_<2, with the outer most edge-channel interfering.

### Observations

**Figure 2:**
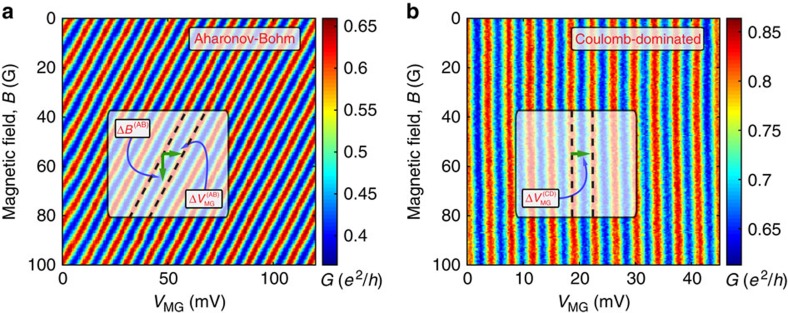
Conductance versus modulation-gate voltage *δV*_MG_ and magnetic field *δ*B measured with Aharonov Bohm dominated and Coulomb-dominated devices. (**a**) 2D conductance measured with the FPI with an Ohmic contact in its centre (seen in Fig. 1b) at *B*=4.7* *T. This plot shows a clear AB behaviour: constant-phase lines are marked with grey lines and the AB periods 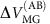 and Δ*B*^(AB)^ are marked accordingly. (**b**) 2D conductance measured with the bare FPI (seen in Fig. 1a) at *B*=4.7 T. This plot shows a clear CD behaviour with no dependence of the conductance on magnetic field; constant-phase lines are marked with grey lines and the CD period 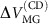 is marked accordingly.

Tuning the magnetic field to the *ν*_B_=2 plateau and partitioning the outer edge channel, conductance oscillations are measured as function of *δB* and *δV*_MG_, with the different FPIs shown in Fig. 1. For the device shown in Fig. 1b, constant-phase lines in the *B*-*V*_MG_ 2D plane follow a negative slope—a typical result in the AB regime (Fig. 2a). A fast Fourier transform (FFT) extracts 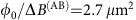
—agreeing well with the lithographically enclosed area, and 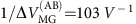
—needed to remove one flux quantum from the interior of the device (equations (1 and 2)). For the device shown in Fig. 1a, no oscillation as function of *δB* is observed—in agreement with the prediction for the CD regime (equations (3 and 4))—with a modulation-gate frequency 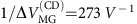
—needed to remove one electron.

**Figure 3:**
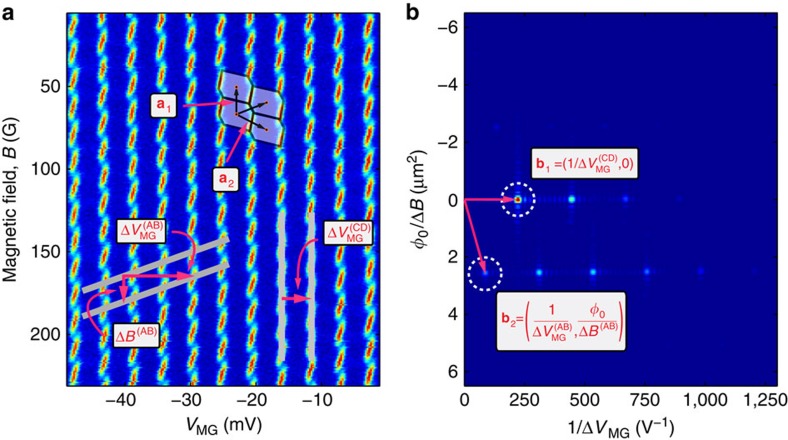
Conductance of a FPI operating in an intermediate regime as a function of the modulation-gate voltage *δV*_MG_ and the magnetic field *δ*B. (**a**) 2D conductance measured with the FPI with an Ohmic contact in its vicinity (seen in Fig. 1c) at *B*=5 T, giving rise to a charge stability diagram. The vectors marked on top of the figure connect different cell and can be translated to physical processes, as elaborated in analysis. Grey lines mark the underlying AB and CD frequencies. (**b**) 2D FFT of the conductance in **a**, revealing a clear lattice structure as well, as expected from an FFT of a lattice. The underlying AB and CD frequencies, marked by two vectors (**b**_**1**_ and **b**_**2**_. respectively, highlighted with white dashed circles), span this Fourier lattice, and the reason for their appearance here is elaborated in analysis.

Turning to employing the device in Fig. 1c, we commenced by measuring the conductance for rather pinched QPCs (<*t*>=0.05), shown in Fig. 3a. This lattice-like conductance plot represents the charge stability diagram of the device and contains information both on the interference and on the device's charging, as we elaborate in Discussion. Although this plot is rather complex, its 2D FFT, shown in Fig. 3b, is simple; it reveals a lattice of peaks (as expected from an FFT of a lattice) all being linear combinations of underlying AB and CD frequencies, denoted on top of the figure. We find 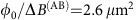 and 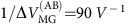 for the AB frequencies, and 1/Δ*B*^(CD)^=0 and 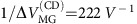 for the CD frequencies. These frequencies are found relatively close to the ones of the ‘pure AB' and ‘pure CD' cases extracted from Fig. 2a,b, respectively. Furthermore, we find 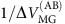 to be the only frequency that depends on magnetic field (in the tesla range), as anticipated from equations (1, 2, 3, 4) (Supplementary Fig. 4).

**Figure 4:**
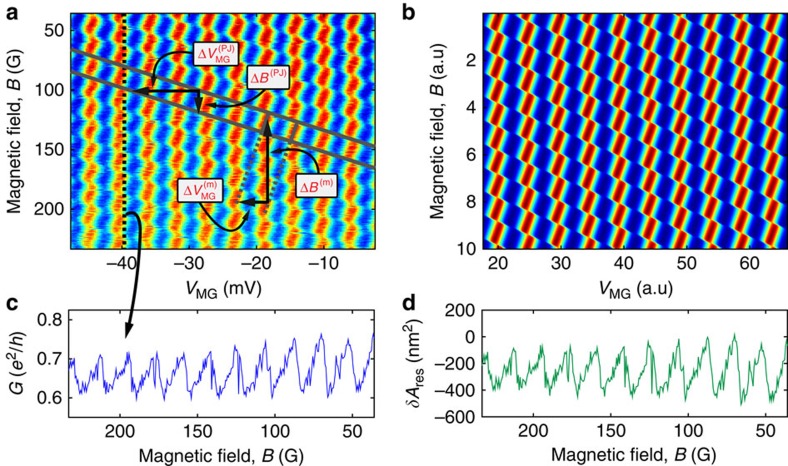
2D conductance plot with clear phase jump lines. (**a**) Conductance is measured with the same device as Fig. 3, but with substantially higher transmission (<*t*>≈0.7). Phase jump lines are marked by solid descending lines and the frequency defined by their slope is marked by 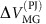 and Δ*B*^(PJ)^. In between adjacent phase jump lines, we have continuous conductance oscillations. By extrapolating these continuous oscillations (dashed ascending lines), we may attribute them periodicities denoted 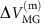 and Δ*B*^(m)^. More details are provided in analysis. (**b**) Theoretical plot, simulated according to the conductance *G* expressed in Discussion. (**c**) Constant *δV*_MG_ line taken from **a**, showing clear phase accumulation followed by abrupt phase jump of 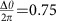, which is also equal to the value of *ξ* (equation (11)). (**d**) The area response *δA*_int_ inferred from the conductance' phase (**c**). The relation between the area response and the phase is obtained by inverting the relation between *δA*_int_ and the conductance *G*, provided in the caption of Fig. 5. Note that indeed (**c**,**d**) are highly similar (as anticipated by the theoretical plots, cf. yellow curves in Fig. 5d,b), but they are not identical. Naturally, for small values of the phase, the conductance is nearly proportional to it.

Moreover, by further opening the confining QPCs to <*t*>=0.7, the presence of abrupt jumps in the conductance (phase jumps) becomes more clear (Fig. 4a), which shows good agreement with the conductance's model that stems from the minimization of the system's energy (Fig. 4b; see Discussion below; Supplementary Notes 2–3; Supplementary Fig. 5–9).

### Analysis

Since the AB and CD types of behaviour are merely two different regimes of operation of the same device, they may be analysed within the same theoretical framework^15^. We address a set-up, where transport through the outer most edge channel at 1<*ν*_B_<2 gives rise to interference. This case has been studied experimentally. We denote the charge added to the lowest Landau level (lowest LL) by *Q*_↓_ , and that added to the upper Landau level (upper LL) by *δQ*_↑_. Hereafter, we refer to the interfering outer most edge channel (compressible ring belonging to the lowest LL) as the ‘edge', and to the rest of the charge inside the FPI as ‘bulk'^23^. The charge variations in the edge and bulk are denoted *δQ*_edge_ and *δQ*_bulk_, respectively. We note that for any device without an Ohmic contact within its centre, *δQ*_↑_ changes discretely accounting for the localized charges in the bulk^15^,^16^.

We consider a minimal capacitive model for describing the FPI set-up^15^,^16^,^24^ depicted in Fig. 1d. The model consists of three capacitances *C*_edge_, *C*_bulk_ and *C*_eb_ describing the mutual Coulomb interactions between the system's three components, *δQ*_edge_, *δQ*_bulk_ and *δV*_MG_. The total energy of the system can then be written as the sum of three contributions^15^,^17^,^25^,^26^, *E*_total_=*E*_eb_+*E*_edge_+*E*_bulk_, where the second and third terms are the edge and bulk charging energies (depending solely on *δQ*_edge_ and *δQ*_bulk_, respectively), and the first term is due to the interaction between them. This first term can be presented as function of their sum *δQ*_edge_+*δQ*_bulk_. The relation between this approach and an earlier one (cf. ref. 15), where the variable *δQ*_edge_ · *δQ*_bulk_ have been employed, is discussed in Supplementary Note 2. The system's charge state is determined by minimizing the total energy *E*_total_.

In the quantum Hall regime, the charge added to the lowest LL, *δQ*_↓_, follows the total flux, *δφ*_tot_, enclosed by the latter: as the flux increases (decreases), single-particle states cross in below (cross in above) the Fermi energy, increasing (decreasing) the number of occupied states:


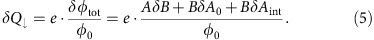


Here, in the third term on the right hand side, *δA*_int_ represents the interaction-induced area response. Thus, the overall variation of the area *δA*=*δA*_0_+*δA*_int_ is separated into two components. The first component is a linear component that is solely a result of variations of the modulation-gate voltage, *δA*_0_=*αBδV*_MG_, and stems solely from its mutual capacitance to the interfering edge. The second component is an oscillating component^7^,^15^, *δA*_int_, which is a function of both *δB* and *δV*_MG_, for which we derive the full expression below (and allowing us to present the total phase as an explicit function of *δB* and *δV*_MG_, cf. equation (8) below).

### Magnetic field variations

As the magnetic field increases and the filling factor decreases, charge is removed from the upper Landau level into the lowest Landau level. More precisely, with every flux quantum added, the degeneracy each Landau level increases by one; resulting in a transfer of a single electron from the upper LL (hence *δQ*_↑_=±1) to the lowest LL (hence *δQ*_↓_=±1), while keeping the total charge in the system constant. The corresponding variation of energy can be expressed in terms of *δQ*_↑_ and *δQ*_↓_^15^,^27^:


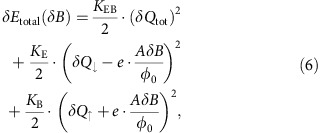


where *δQ*_tot_=*δQ*_↓_+*δQ*_↑_, 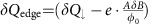, 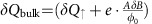 and all three coefficients are related to the relevant different capacitances^15^: 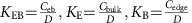 are system specific, with *D*=(*C*_edge_+*C*_eb_)(*C*_bulk_+*C*_eb_)−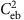.

Equation (6) implies that the energy is minimized if 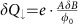 and 
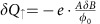
, leading to *δQ*_tot_=0 at all times. This continuous charging of the edge, *δQ*_↓_, should result in the periodicity Δ*B*=*φ*_0_/*A*. The latter coincides with the behaviour in the AB regime, as seen in Fig. 2a, measured with the device shown in Fig. 1b. In this device, while *δQ*_↓_ is continuously charged from the leads, *δQ*_↑_ is discharged into the centre Ohmic contact.

**Figure 5:**
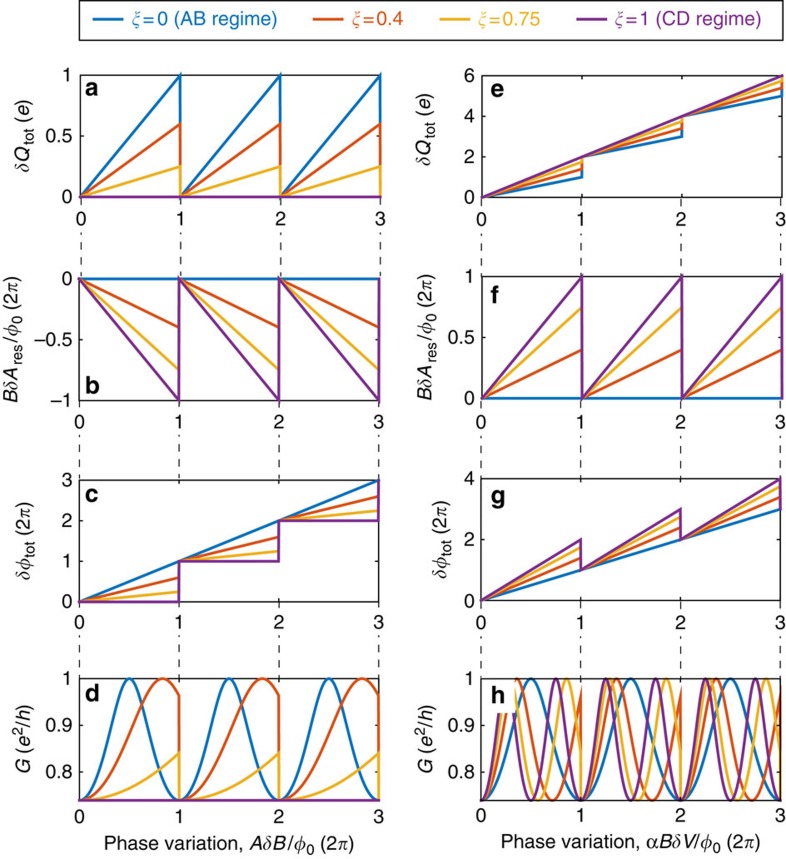
Theoretical plots for the Fabry–Pérot interferometer in all regimes. (**a**) Total charge in the system, *δQ*_tot_=*δQ*_↓_+*δQ*_↑_. (**b**) The area response *δA*_int_. (**c**) The total flux *δφ*_tot_, including both the externally varied phase *AδB*/*φ*_0_ and the response *BδA*_int_/*φ*_0_. (**d**) The resulting conductance for relatively open QPCs (*t*^2^=0.85), taking the straightforward form 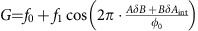 (further details are provided in Supplementary Note 3). All four graphs (**a**–**d**) are plotted versus variations of the magnetic field. (**e**–**h**) Similar graphs as **a**–**d**, as a function of modulation-gate voltage *δV*_MG_. The plots are given for the simple case of *ν*_B_=2. For simplicity, we plot **e**–**h** in the case *C*_bulk_=*C*_edge_ at bulk filling factor *ν*_B_=2, leading to a CD frequency that is twice of the AB frequency. The choice of these values does not change the qualitative picture, and we note that we find *C*_bulk_=1.45̇*C*_edge_ in the experiment, deduced from the measured frequencies.

Previous measurements have also reported the observations of AB oscillations even without the centre Ohmic contact, but rather with a top gate covering the device^7^,^8^. In that case, the upper LL's charge *δQ*_↑_ cannot vary continuously (as in the case with an Ohmic contact at the centre of the device), but rather discretely, because the upper LL is isolated by an incompressible strip^15^,^23^. Thus, as the magnetic field increases such that *AδB*<*φ*_0_, the lowest LL is continuously charged by 
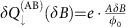
, leading to an increase of the total charge 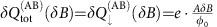 with *δQ*_↑_=0 due to the upper LL's charge quantization. This of course will increase the charging energy (which favors *δQ*_tot_=0; first term in equation (6)), which is possible since the top gate effectively decreases *K*_EB_ (and thus *C*_eb_), such that *K*_EB_<<*K*_E_ (and *C*_eb_<<*C*_bulk_)^7^,^8^,^15^. Then, once a whole flux quantum is added *AδB*=*φ*_0_, the energy can again be minimized by discharging the upper LL abruptly *δQ*_↑_=−*e*. This AB behaviour is summarized in Fig. 5a–c (blue), leading to a conductance depicted in Fig. 5d (blue).

On the other hand, as alluded above in the CD regime, the total charge must be constant 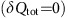
at all times due to the large charging energy *K*_EB_≫*K*_E_, thus *C*_eb_≫*C*_bulk_ (in the absence of an Ohmic contact or top gate). As a result, when the applied magnetic field is increased, the area shrinks to prevent the enclosed flux from increasing. This means 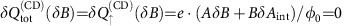, with *δQ*_↓_=0 due to the bulk's quantization, leading to *δA*_int_=−*AδB*/*B*. In that case, as the interferometer's area shrinks, a dipole is created: a depleted region (positively charged) is created just outside the boundary of the shrinking interferometer, while an excess negative charge accumulates inside. The energy increase due to this dipole is reflected by the second and third terms in equation (6). Once a whole flux quantum is added *AδB*=*φ*_0_, the energy can again be minimized by abruptly moving an electron from the upper LL to the lowest LL, described by the simultaneous variation *δQ*_↑_=−*e* and *δQ*_↓_=+*e*. At this point, the area response *δA*_int_ should abruptly vanish from ∼810 nm^2^ (a flux quantum's area at 5 T) to 0 μm^2^. Such an abrupt response cannot be inferred from the measured conductance (Fig. 2b), since it should result in a 2*π* variation of the Aharonov–Bohm phase. This leaves the conductance constant as function of *δB*. This CD behaviour is summarized in Fig. 5a–c (purple), leading to a conductance depicted in Fig. 5d (purple).

To summarize, for any device without an Ohmic contact within its centre (implying a quantization condition on *δQ*_↑_), the system ‘decides' whether it shrinks or not, and by how much, according to the values of *K*_EB_ and *K*_E_. Taking the derivative of the total energy with respect to *δA*_int_, we obtain *δA*_int_=−*ξ*·*AδB*/*B*, with 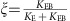. The AB regime corresponds to the limit *K*_E_≫*K*_EB_, leading to *ξ*=0 and *δA*_int_=0; the CD corresponds to the limit *K*_E_<<*K*_EB_, leading to *ξ*=1 and *δA*_int_=−*AδB*/*B*. The behaviour in the intermediate regime is summarized in Fig. 5a–c (red and yellow for *ξ*=0.25 and *ξ*=0.75, respectively), resulting in the conductance depicted in Fig. 5d.

The theoretical prediction (*ξ*=0.75; Fig. 5d, yellow) is consistent with the measured conductance shown in Fig. 4c, from which we have retrieved both the area=2.6 μm^2^ (from the main periodicity) and the interaction parameter *ξ*=0.75 (from the amplitude of the phase jumps). The possibility to extract these two parameters from a single magnetic field scan demonstrates the versatile information contained in the intermediate regime—it incorporates both interactions (quantified by *ξ*) and interference (related to *A*). Furthermore, from the conductance (Fig. 4c), we infer the evolution of the area response, *δA*_int_, as function of *δB*, shown in Fig. 4d. We find a maximal area response of *δA*_int_=500 nm^2^, which is in good agreement with our expectation 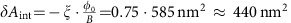.

### Modulation-gate variations

The two handles we have, varying the magnetic field *δB* and varying the modulation-gate voltage *δV*_MG_, are substantially different from each other. While in both AB and CD regimes, the total charge must be conserved for *δB* variations on the scale of several flux quanta, *AδB*≫*φ*_0_, *δV*_MG_ induces charge on the device through its capacitance *γ*: applying *δV*_MG_ requires charging the device by *δQ*_tot_=*γδV*_MG_ that divides into each Landau levels^15^,^16^: 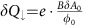 and 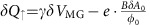. Thus, the energy variation of the system (equation (6)) can be reformulated as:


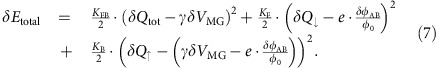


Although equation (7) is valid for any variation of *δB* and *δV*_MG_, only modulation-gate variations, *δV*_MG_, are considered in this section.

In the AB regime, as the modulation-gate voltage increases by *δV*_MG_ such that 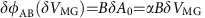, the lowest LL is continuously charged by 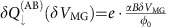 allowing us to observe Aharonov–Bohm interference with a period 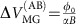 (cf. Fig. 2a). Note that *α* is independent to the magnetic field (Supplementary Fig. 4). Simultaneously, we expect the upper LL to be continuously charged from the centre Ohmic contact, satisfying 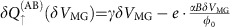. Alternatively, with top-gated devices (*K*_EB_<<*K*_E_), the upper LL acquires an additional electron discretely only once this last expression (which can be regarded as the induced charge in the bulk) amounts to an increase by a whole-electron charge 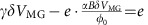. This AB behaviour is summarized in Fig. 5e–g (blue) and results in a conductance depicted in Fig. 5h (blue).

By contrast, in the CD regime, the high charging energy *K*_EB_<<*K*_E_ requires 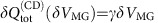 for all values of *δV*_MG_. As *δQ*_↑_=0 due to charge quantization in the upper LL, it follows that 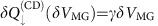. According to equation (5), this necessitates an area response 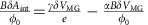, (which is always larger than zero since 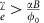; see Supplementary Figs 2 and 4). Namely, while in the AB regime the area dilations are dictated solely by the mutual capacitance between the edge and the modulation gate, *α*, here, in the CD regime, the area dilations overshoots to satisfy the capacitance of the device (including both the edge and the bulk) to the modulation gate, *γ*. This overshoot will cost an energy proportional to *K*_E_ (equation (7)). Then again, once this overshoot is equivalent to a whole electron, the area will retract back minimizing the total energy. This CD behaviour is summarized in Fig. 5e–g (purple) and results in a conductance depicted in Fig. 5h (purple).

Similarly to the analysis of magnetic field variations, we can express the area response to variations of *δV*_MG_ for any value of the interaction parameter *ξ*: we obtain 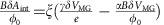 resulting in a total area variation 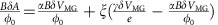. This is summarized in Fig. 5e–h (red and yellow for *ξ*=0.25 and *ξ*=0.75, respectively).

### Analysis of the entire ***B***−***V***_**MG**_ plane

So far, we have analysed the area response to variations of either *δB* or *δV*_MG_. We now turn our attention to concomitant variations of both. This will allow us to analyse the data shown in Figs 3a and 4a. Notably, the interference pattern in Figs 3a and 4a may be decomposed into two ingredients. First, descending lines along which phase jumps take place (cf. solid lines in Fig. 4a). The respective periodicities are denoted 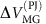 and Δ*B*^(PJ)^ (marked on top of Fig. 4a, with the superscript ‘PJ' for ‘phase jump'). Second, continuous conductance oscillations with respect to modulation-gate voltage and magnetic field in between two adjacent phase jump lines. The corresponding periodicities are then 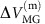 and Δ*B*^(m)^,respectively (marked on top of Fig. 4a as well, with the superscript ‘m' for ‘modified'). These continuous oscillations reflect the interference of electrons at the edge. Crossing a maximal conductance line (for example, following arrow **a**_**2**_ in Fig. 3a) corresponds to an incremental variation of *δQ*_↓_ by ±*e*. Here, ‘modified' alludes to the fact that these continuous oscillations are in fact coherent AB oscillations modified by the Coulomb interaction. Likewise, crossing a phase jump line (for example, following arrow **a**_**1**_ in Fig. 3a) implies a change of the upper LL's charge *δQ*_↑_ by ±*e*. Naturally, the combination of these two types of processes gives rise to the system's charge stability diagram in the *B*−*V*_*MG*_ plane.

To express the modified periodicities Δ*B*^(m)^ and 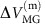 (in between abrupt changes of the charge), we need to determine an expression for the interference phase (equation (5)) for a fixed number of electrons in the upper LL. We first generalize the dependence of the area response on *δB* and *δV*_MG_ by combining the results obtained in the previous sections: 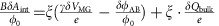, where the last term represents phase jumps that accompany variations of *δQ*_↑_=±*e* (for further details see Supplementary Notes 2–3). This result is then plugged into the expression for the total phase (equation (5))^15^:


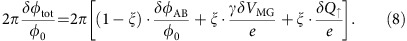


Note that the last term of the of the equation accounts for abrupt phase jumps. The respective modified periodicities are:


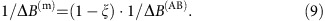



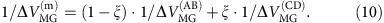


These two frequencies are notably a linear combination of the AB and CD frequencies (cf. equations (1, 2, 3, 4)): 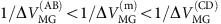 and 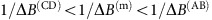. Constant flux lines of *δφ*_tot_ follow a negative slope in the *B*−*V*_MG_ plane, similar to the pure AB case. We extract Δ*B*^(m)^=65.9 G and 
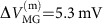
 from our data; each of these periodicities leads to an interaction parameter *ξ*=0.75±0.01. The periodicities associated with phase jumps are found to be 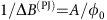 and 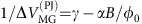 (cf. equations (1, 2, 3, 4); see Supplementary Note 3 for details). We find this relation to be in agreement with the measured values. Note that these periodicities do not depend on the value of *ξ*.

Combining the modified periodicities (equations (9 and 10)) with the phase jump periodicities allows us to construct the charge stability diagram. Naturally, vectors connecting different cells in the diagram (Fig. 3a) represent different discrete charge variations (*δQ*_↓_,*δQ*_↑_)=(*n*,*m*) (cf. Supplementary Note 2). It is convenient to select the following basis (Fig. 3a): 
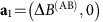
 and 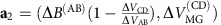. Here, **a**_1_ represents the process of moving one electron from the upper to the lowest LL (namely, *n*=1 and *m*=−1, as explained in ‘magnetic field variations'), while **a**_2_ represents adding one electron to the lowest LL and keeping the upper LL's charge constant (namely, *n*=1 and *m*=0). Evidently, their sum **a**_1_+**a**_2_ refers to adding one electron to the upper LL, while keeping the lowest LL's charge constant. The reciprocal lattice is spanned by the pure AB and CD frequencies, 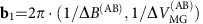 and 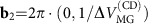 respectively (cf. Fig. 3b).

### Phase jumps and proposed experiments

Finally, we study qualitatively the phase jump taking place when a phase jump line is crossed, following an infinitesimal variation of either *δB* or *δV*_MG_. The acquired phase is given by^15^:


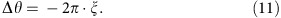


Evidently, in the extreme AB and CD cases this phase jump is zero (*ξ*=0) or unobservable (*ξ*=1), respectively. Experimentally, the value of *ξ* (0<*ξ*<1) can be deduced from directly measuring the phase jump (seen in Fig. 4c). We find *ξ*≈0.75 (similar to the value we obtained independently by comparing the measured frequencies with equations (9 and 10) of the previous section). This value of *ξ* corresponds to an area jump of 440 nm^2^ (cf. Fig. 4d).

A possible extension of our analysis to the FQHE regime is straightforward and results in a phase jump given by:


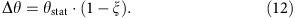


where *θ*_stat_ is the quasi-particles' statistical braiding phase (for example, 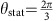 for anyons at *ν*_B_=1/3). While the difference between equation (11) and equation (12) is seemingly minute, its impact might be significant. It is apparent that, by probing Δ*θ* in a fractional filling factor, the requirement for probing *θ*_stat_ is 0≤*ξ*<1. This requirement is to be compared with previous measurements that focused on a pure AB, *ξ*=0 and pure CD, *ξ*=1 (anyonic statistics cannot be probed). Breaking this dichotomy between the pure AB and pure CD regimes thus provides us with a toolbox to probe anyonic statistics.

We stress that the protocol to probe fractional statistics requires the study of the conductance as function of the magnetic field and the modulation-gate voltage (similarly to Figs 3a and 4a). This is needed to identify the regime of operation (the parameter *ξ*) with no ambiguity. Previous reports^28^,^29^ presenting conductance oscillations in fractional filling factors have failed to show such 2D scans of the conductance in a 2D parameter space, and were thus criticized for not excluding a CD behaviour, or for being otherwise inconclusive^8^,^19^,^30^. Other works, which did identify the regime of operation, simply failed to obtain an AB behaviour in fractional filling factors. These attempts utilized both large area and small area devices. For the former, Coulomb effects are expected to be negligible if a top gate is placed on top of the sample^7^,^8^ or an Ohmic contact is placed at the centre of the device^22^, rendering the regime of operation AB. Yet an AB interference pattern (observable in the integer quantum Hall effect regime) has not been observed for fractional filling factors. This might be attributed to a short coherence length of anyons in such relatively large devices. One may then resort to smaller FPIs. Unfortunately, reducing the device's size leads to the undesired CD regime in the case of top-gated devices^7^,^8^, and, in the case of devices with Ohmic contacts at the centre, further miniaturization is limited. The device reported here, having an Ohmic contact placed outside its circumference, operates in a regime intermediate between AB and CD. Given the possibility to make the device's size small in such a design, yet maintaining *ξ*<1 with a clear signature of coherent Aharonov–Bohm oscillations, presents us with an intriguing prospects: notwithstanding a short quasi-particle coherence length, coherency may still be maintained over the size of the interferometer. Interference signal, a smoking gun evidence for anyonic statistics, is then potentially observable.

## Discussion

We provide here an experimental evidence of area modulations of a FPI implemented in the quantum Hall effect regime. These modulations stem from the minimization of the system's energy, as the applied magnetic field and the modulation-gate voltage are varied. Our analysis does not rely on the coherent operation of the device. The measurement of the area modulations, though, could be realized through the observation of coherent interference oscillations. The area evolution consists of a continuous shrinking when increasing magnetic field, followed by an abrupt dilation.

We have employed a theoretical framework that accounts for both the AB and the CD, and a novel intermediate regime. The latter enables us to construct the system's charge stability diagram as well, showing good agreement with a minimal theoretical model. Both the area breathing and the charge stability diagram are used towards a complete characterization of the device, the latter being hidden in the pure AB or CD limits.

Utilizing a similar interferometer operating in the intermediate regime in the domain of the FQHE regime is called for. The novelty of our device lies within the possibility to explore a wide range of the interaction parameter *ξ*, in which the quasi-particles' braiding statistics is observable. In other words, our findings reported here pave a new path for probing anyonic statistics in the FQHE regime.

## Methods

### Ohmic contacts

As explained in the text, the ‘pure' CD regime can be avoided through undermining the effect of Coulomb interactions. This can be achieved utilizing two different methods:

First: we place a grounded Ohmic contact inside the interferometer's bulk, thus avoiding its charge quantization (cf. the device in Fig. 1b). The Ohmic contact consists of 106 nm Au/53 nm Ge/40 nm Ni alloyed to the heterostructure having a resistance of 500 Ω at zero magnetic field. This Ohmic contact is not resistively coupled to the edge (the observed coherent phase oscillations are evidence to that). This important point has been verified in a previous work (cf. Supplementary Fig. 9 in ref. 22), employing a more complex set-up. The latter consisted of a FPI with a centre contact and an additional QPC placed along the interferometer's edge, allowing to reflect edge channels selectively into the centre Ohmic contact. Then, by measuring the current at the centre contact, we have verified that it is non-vanishing only once the chiral channel corresponding to the LLL is physically reflected into this contact.

Second: this consists of lowering the device's charging energy, *K*_EB_. For this purpose, two schemes are available. One scheme consists of covering the whole area of the FPI by a metallic top gate, increasing its capacitance, thus lowering its charging energy^7^,^8^. This scheme has not been utilized in our measurements, but has been reported to work well for relatively large FPIs (areas >12 μm^2^)^7^,^8^, resulting in a ‘pure' AB behaviour. On the other hand, devices of area <4 μm^2^ have been found to operate in the CD regime, even if covered by top gate^7^,^8^. This was attributed to the different scaling of the capacitances *C*_eb_ and *C*_bulk_ with area^15^. The other scheme relies on placing an Ohmic contact in the vicinity of the interferometer. The role of the Ohmic contact here can be understood through a mutual capacitance *C*_ob_ between the Ohmic contact and the bulk, similar to the effect of top gates^7^,^8^; we find, though, that this placement of an Ohmic contact is substantially more effective than a top gate. The mutual capacitance effectively increases the capacitance of the bulk (*C*_bulk_→*C*_bulk_+*C*_ob_), accounting for the partial screening by the Ohmic contact, while keeping the capacitance between the edge and the bulk unchanged (*C*_eb_→*C*_eb_). This leads to a decrease of the interaction parameter, *ξ* (*ξ*=0.75 in the results reported here, compared with *ξ*=1 with top-gated devices of the same size 4 μm^2^). As is discussed in the main text, this new design gives rise to the intermediate regime.

### Measurement techniques

Our electronic set-up for conductance measurements is depicted in the Fig. 1c. We note that two amplifiers were used in the course of our measurements; the first, a homemade voltage preamplifier at *T*=1 K having a gain factor ∼10 and a commercial amplifier (NF SA-220F5) at room temperature having a voltage gain ∼200.

All experiments are performed within ^3^He–^4^He dilution fridges. Electron temperatures are measured via shot-noise measurements by driving a variable d.c. source current at a centre frequency of 800 kHz and bandwidth of 10 kHz.

### Data availability

The data that support the findings of this study are available from the corresponding author upon request.

## Additional information

**How to cite this article:** Sivan, I. *et al.* Observation of interaction-induced modulations of a quantum hall liquid's area. *Nat. Commun.* 7:12184 doi: 10.1038/ncomms12184 (2016).

## Supplementary Material

Supplementary Information Supplementary Figures 1-9, Supplementary Notes 1-3 and Supplementary References
