# Supplementary material for: Observation of interaction-induced modulations of a quantum Hall liquid's area
Source: Nat Commun. 2016 Jul 11;7:12184. doi: 10.1038/ncomms12184 (PMC4942580; doi:10.1038/ncomms12184)
Supplement: Supplementary Information — Supplementary Figures 1-9, Supplementary Notes 1-3 and Supplementary References [file ncomms12184-s1.pdf]

Supplementary Figures

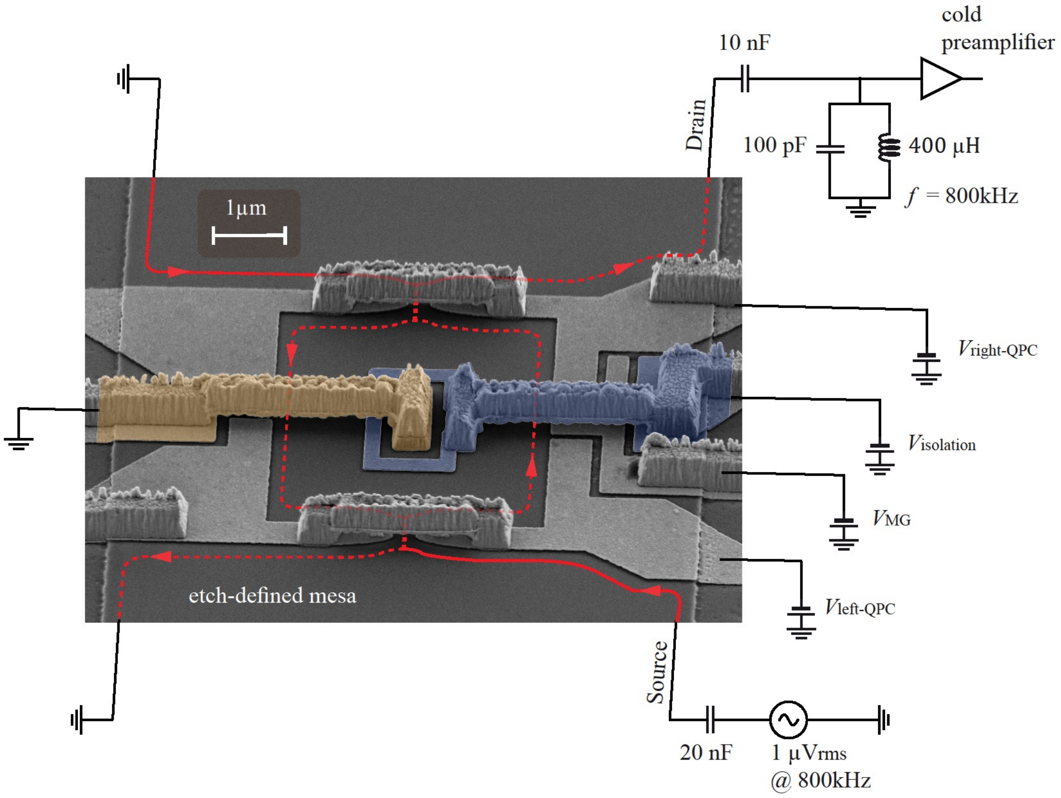

Supplementary Figure 1 | SEM images and illustration of a FPI with a center ohmic contact surrounded by an isolation-gate. The regime of this device can be tuned by varying the voltage on the isolation gate $V_{\text{isolation}}$; it can be tune to either the pure AB regime, by setting $V_{\text{isolation}} = 0$, or to an intermediate regime by setting a low enough voltage resulting in depletion of the electron-gas below it. We note that a pure CD regime cannot be reached with this device.

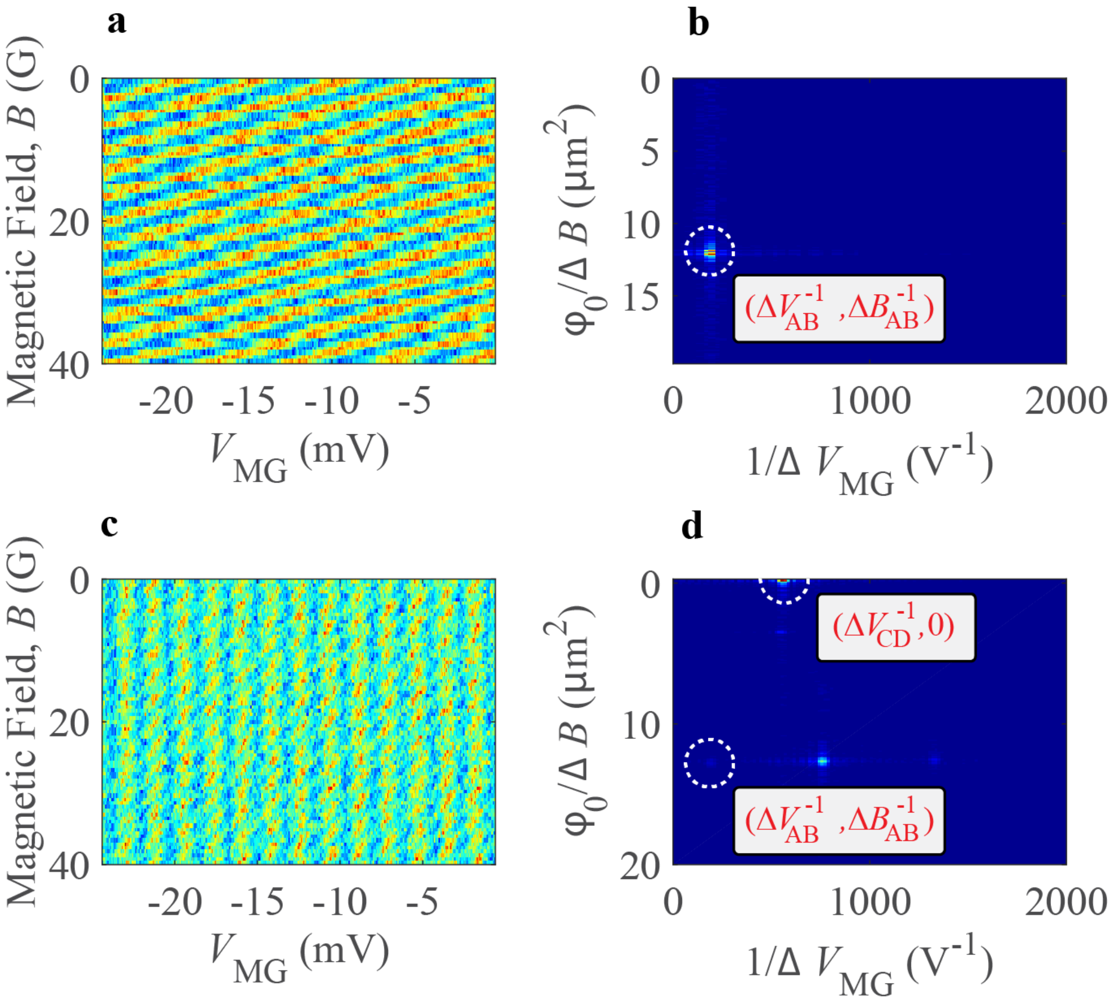

Supplementary Figure 2 | Conductance measurements as a function of the modulation-gate voltage V_{MG} and magnetic field B, measured with the 12 μm^2 device with an isolation-gate around its center ohmic-contact shown in Fig. 1d. **a**, 2D conductance measured while the isolation-gate is unbiased. This plot shows clear AB behavior. **b**, FFT for **a**; A single frequency of the AB is observed, in agreement with the Eq. 1 & 2 in the main text. **c**, 2D conductance measured while the isolation-gate is biased so that the 2DEG beneath it is depleted. **d**, FFT for **c**; several frequencies are observed, all being linear combinations of the AB and CD frequencies. We note that frequency of the AB in the two plots takes the same value, as anticipated. Dependence of these frequencies on magnetic field is detailed in Supplementary Figure 3.

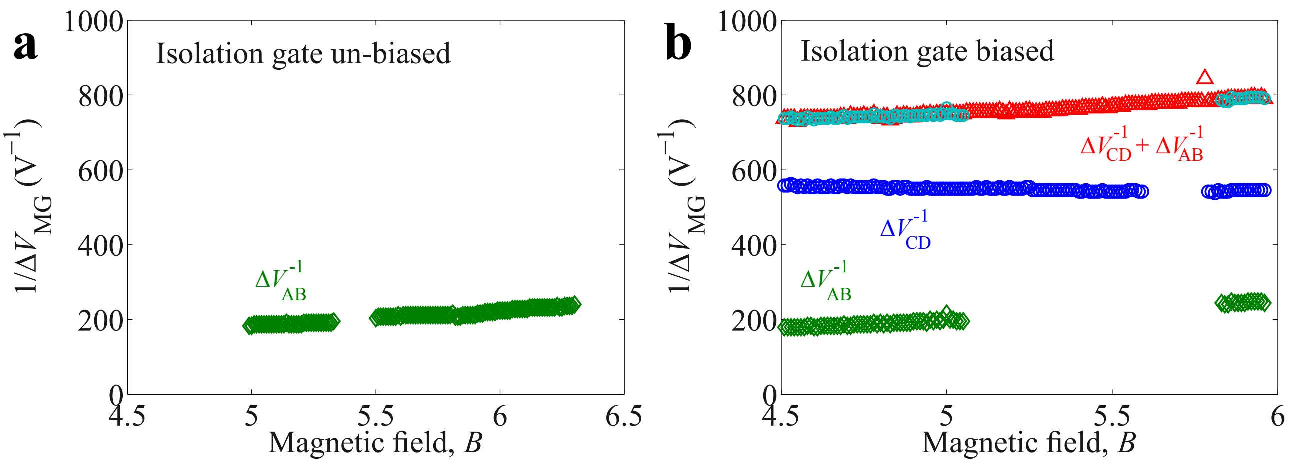

Supplementary Figure 3 | Aharonov-Bohm and Coulomb-dominated frequencies ($1/\Delta V_{\mathbf{MG}}^{(\mathbf{AB})}$ and $1/\Delta V_{\mathbf{MG}}^{(\mathbf{CD})}$) as a function magnetic field, measured with the $12\ \mu m^2$ device with an isolation-gate around its center ohmic contact shown in Supplementary Figure 1. a, AB frequency measured while the isolation gate is unbiased. **b**, AB and CD frequencies measured while the isolation-gate is pinched-off, giving rise to the intermediate regime. The AB frequency (green, diamond) is easily distinguished according to the graph in **a**. The CD frequency is distinguished by its independence on magnetic-field according to Eq. 3 & 4 in the main text. The third measured frequency (red, triangles) coincides with the some of the two (cyan, circles).

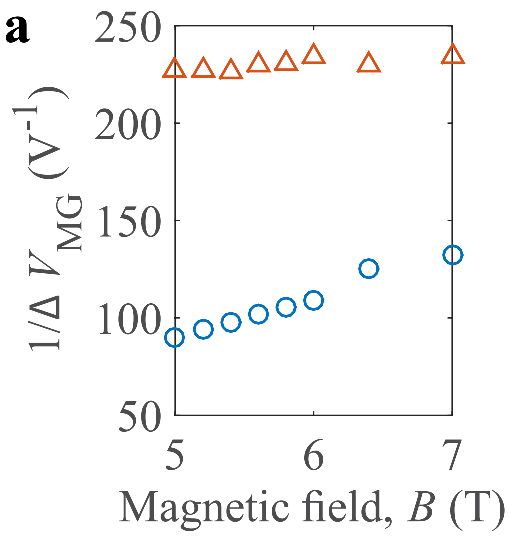

Supplementary Figure 4 | Aharonov-Bohm and Coulomb-dominated frequencies ($1/\Delta V_{MG}^{(AB)}$ and $1/\Delta V_{MG}^{(CD)}$) as function magnetic field, measured with the $2.6\ \mu m^2$ device with a center Ohmic contact in its center, shown in Supplementary Figure 1. a, Modulation-gate frequencies with respect to magnetic field; AB frequency $1/\Delta V_{MG}^{(AB)}$ (blue, circles) and CD frequency $1/\Delta V_{MG}^{(CD)}$ (red, triangles). While the first depends linearly on magnetic field, the second doesn't, as expected according to Eq. 1 & 2. **b**, Magnetic field frequencies (corresponding to periods in the Gauss scale) with respect to magnetic field (in the Tesla scale); AB frequency $1/\Delta B_{MG}^{(AB)}$ (blue, circles) and CD frequency $1/\Delta V_{MG}^{(CD)}$ (red, triangles). While the first has a finite value representing the device's area, the second doesn't; both of the frequencies do not show a dependence linearly on magnetic field, as expected according to Eq. 1- 4 in the main text.

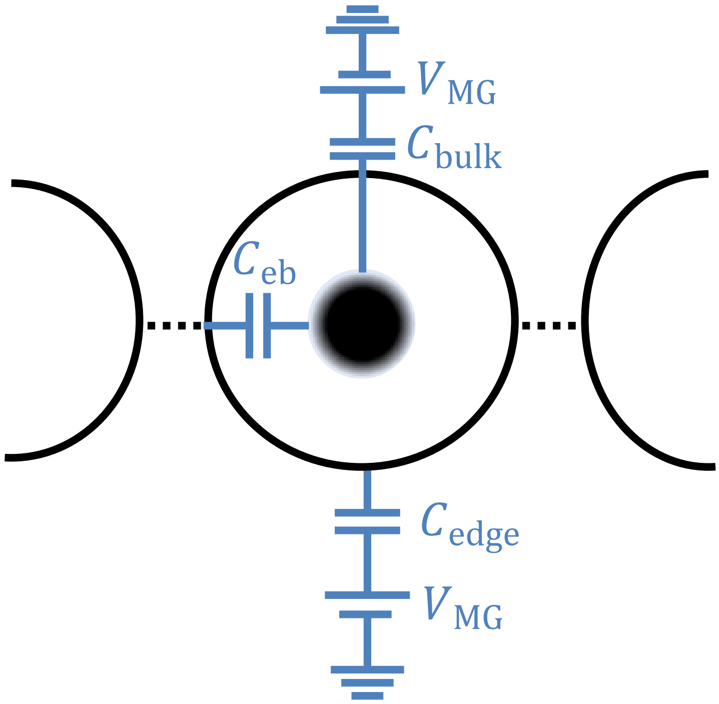

Supplementary Figure 5 | A Fabry-Perot interferometer at filling factor $1 < \nu_B < 2$. The system consists of an edge of the $\nu_B = 1$ Landau level and a compressible puddle of charge in the bulk, separated by an incompressible $\nu = 1$ region. It is effectively modeled by three capacitors, $C_{\mathrm{edge}}, C_{\mathrm{bulk}}, C_{\mathrm{eb}}$, describing the electrostatic energy due to charging at the edge, at the bulk, and due to edge-bulk interaction.

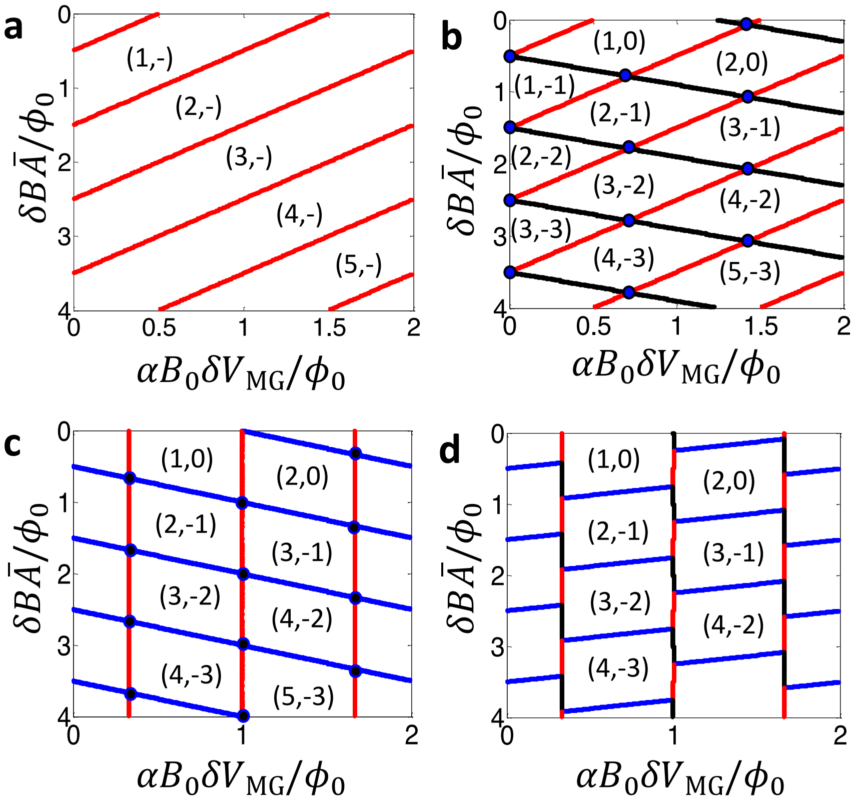

Supplementary Figure 6 | Charge stability diagrams of the FPI in the ($\boldsymbol{\delta B}, \boldsymbol{\delta V}_{\mathbf{MG}}$) plane. a, **b**, **c**, **d**, Charge stability diagrams for scenario (i), (ii), (iii) and (iv) respectively. The numbers in parentheses indicate the charge state ($\delta N_{\downarrow}, \delta N_{\uparrow}$). Note that in **a**, the number of electrons in the compressible puddle is not well-defined (no requirement for charge neutrality). Here *red lines* represent degeneracy between ($\delta N_{\downarrow}, \delta N_{\uparrow}$) and ($\delta N_{\downarrow} + 1, \delta N_{\uparrow}$), corresponding to conductance peaks. *Blue lines* represent edge-bulk charge reshuffling (inducing a phase jumps), i.e., $(\delta N_{\downarrow}, \delta N_{\uparrow}) \rightarrow (\delta N_{\downarrow} + 1, \delta N_{\uparrow} - 1)$. *Black lines* represent degeneracy between ($\delta N_{\downarrow}, \delta N_{\uparrow}$) and ($\delta N_{\downarrow}, \delta N_{\uparrow} + 1$). In **b**, $\frac{K_{\mathrm{B}}}{K_{\mathrm{E}}} = 2.5$ and $\frac{K_{\mathrm{EB}}}{K_{\mathrm{E}}} = 0$, in **c**, $\frac{K_{\mathrm{B}}}{K_{\mathrm{EB}}} = 1.5$ and $\frac{K_{\mathrm{E}}}{K_{\mathrm{EB}}} = 0$, and in **d**, $\frac{K_E}{K_{EB}} = \frac{K_{\mathrm{B}}}{K_{\mathrm{EB}}} = 0.001$. In all cases, $\frac{\gamma \phi_0}{\alpha B_0 |e|} = 1.5$. We did not constrain the values of K_{E} and K_{B} depending on capacitances, taking into account single particle energy level spacing in reality.

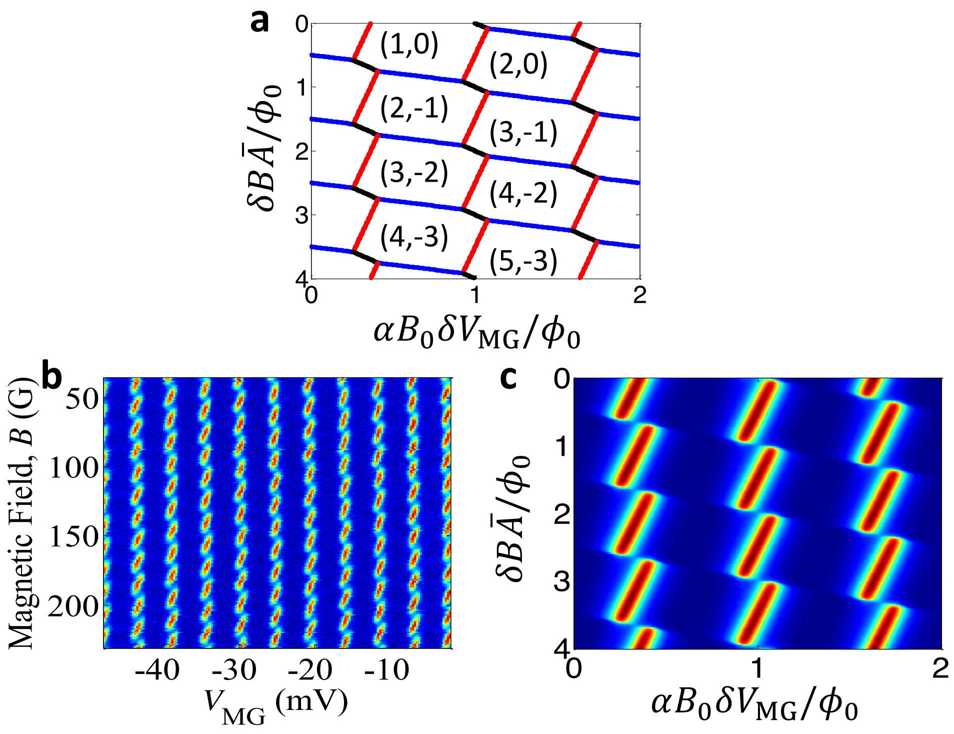

Supplementary Figure 7 | a, Stability diagram of the QD in the intermediate regime (between the Aharonov-Bohm regimes and Coulomb-dominated regimes). Conductance is shown in the $(\delta B, \delta V_{MG})$ plane in **b**, experiment and **c**, theory based on our capacitive model. In **a** and **c**, we use parameters $\frac{K_{\mathrm{B}}}{K_{\mathrm{E}}} = 7$, $\frac{K_{EB}}{K_E} = 3$, and $\frac{\gamma\phi_0}{\alpha B_0|e|} = 1.5$. In **c**, $\frac{\Gamma_{\mathrm{bulk}}}{K_{\mathrm{E}}} = 0.0125$ and $R_{\mathrm{L}} = R_{\mathrm{R}} = 0.6$.

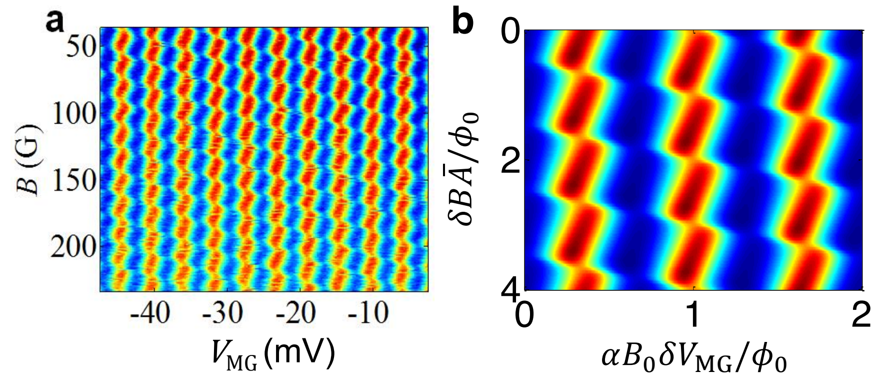

Supplementary Figure 8 | The conductance in the $(\delta B, \delta V_{\mathrm{MG}})$ plane in **a**, experiment and **b**, theory. In **b**, we use parameters $\frac{\kappa_{\mathrm{B}}}{\kappa_{\mathrm{E}}} = 7$, $\frac{\kappa_{\mathrm{EB}}}{\kappa_{\mathrm{E}}} = 3$, $\frac{\gamma\phi_0}{\alpha B_0|e|} = 1.5$, $\frac{\Gamma_{\mathrm{bulk}}}{\kappa_{\mathrm{E}}} = 0.375$ and $R_{\mathrm{L}} = R_{\mathrm{R}} = 0.2$.

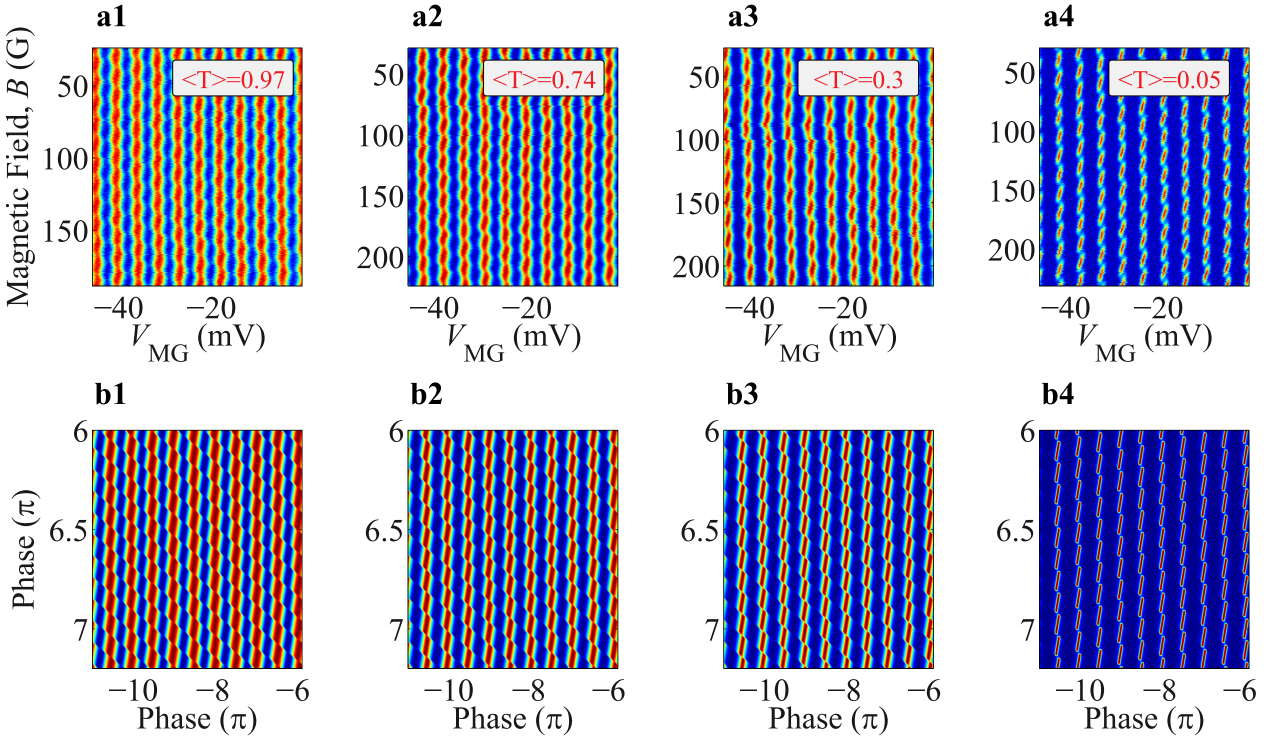

Supplementary Figure 9 | Effect of the transmission on the modified-AB pajama. We shows the evolution of the pajama plot as the transmission of the FPI's QPCs are being increased. Noticeably, even when scanning the whole range of transmission from $<T>= 0.05$ to $<T>= 0.97$ the qualitative picture remains. Specifically we stress that the modified-AB frequencies do not change over this range, keeping the values given in Fig. 4a in the main text. **a1-a4**, Measured conductance as function of the modulation-gate voltage V_{MG} and magnetic-field B for different values of the average transmission. **b1-b4**, Calculations of the conductance oscillations (for details see Supplementary Note 3).

Supplementary Notes

Supplementary Note 1. Fabry-Perot interferometer (FPI) with a center Ohmic-contact surrounded by gate

An additional type of interferometer was found to show an intermediate regime between pure Aharonov-Bohm (AB) and Coulomb Dominated (CD). This interferometer, shown in Supplementary Figure 1 has an ohmic contact inside its bulk, which is surrounded by an additional gate. Supplementary Figures 2a,c show conductance measurements with respect to magnetic field and modulation gate (MG). The first conductance measurement was performed while the isolation-gate around the center ohmic-contact was unbiased; giving rise to simple AB oscillations (pajama-like stripe) following Eq. 1 & 2 in the main text (Supplementary Figures 2a). Its FFT reveals a single 2D-frequency with $\phi_0/\Delta B^{(\mathrm{AB})} = 12.7\ \mu\mathrm{m}^2$ and $\Delta V_{\mathrm{MG}}^{(\mathrm{AB})} = 4.9$ mV (Supplementary Figure 2b). The second (Supplementary Figure 2c) was performed while the isolation-gate was negatively biased so that the 2DEG beneath it is depleted (Supplementary Figure 2c). This graph incorporates several frequencies, seen in Supplementary Figure 2d, all being linear combinations of AB and CD frequencies, similarly to Fig. 3b in the main text. The AB frequency in this configuration is the same as the one in Supplementary Figures 2a and the additional CD frequency is $\Delta V_{\mathrm{MG}}^{(\mathrm{CD})} = 1.7$ mV

The underlying frequencies $\Delta V_{\mathrm{MG}}^{(\mathrm{AB})}$ and $\Delta V_{\mathrm{MG}}^{(\mathrm{CD})}$ clearly differ by their dependence and independence (respectively) on the magnetic field (see Eq. 1-4 in the main text). In Supplementary Figure 3 we show their dependence on magnetic field, measured while the isolation-gate was un-biased (Supplementary Figure 3a) and biased (Supplementary Figure 3b). The $1/\Delta V_{\mathrm{MG}}^{(\mathrm{AB})}$ (green, diamond) frequency is similar in both graphs, showing a clear linear dependence on the magnetic field, in agreement with Eq. 1 & 2 in the main text. The $1/\Delta V_{\mathrm{MG}}^{(\mathrm{CD})}$ frequency (blue, triangles) is recognized by its independence on magnetic field according to Eq. 3 & 4 in the main text. Then, the third frequency (red, triangle), coincides with the sum of the two elementary ones $1/\Delta V_{\mathrm{MG}}^{(\mathrm{CD})} + 1/\Delta V_{\mathrm{MG}}^{(\mathrm{AB})}$ (cyan, circles).

Supplementary Note 2. Capacitive model of the FPI

A capacitive model for the FPI

The FPI setup is depicted in Supplementary Figures 5 as an electrical circuit. By means of two QPCs, a quantum Hall (QH) strip is separated into (weakly coupled) three parts: left and right leads, and the FPI in the middle which can be regarded as a quantum dot (QD). As in the main text, we consider the

case of filling factor $1 < \nu_{\mathrm{B}} < 2$. The FPI consists of an outer edge channel (with a chiral one-dimensional model), and an interior compressible puddle in the bulk[2], separated by an incompressible $\nu = 1$ electron gas.

We consider a capacitive model, described by an equivalent electrostatic circuit, depicted in Supplementary Figure 5, which is widely accepted to describe our setup[1,3,4]. It consists of three effective capacitors, $C_{\mathrm{edge}}, C_{\mathrm{bulk}}, C_{\mathrm{eb}}$, describing the electrostatic energy due to charging at the edge channel, at the bulk, and due to edge-bulk interaction, respectively. The charge distribution at the edge and the bulk is dictated, in principle, by the varied perpendicular magnetic field δB, and the modulation gate δV_{MG}, and is subject to minimization of the electrostatic energy.

We first consider an initial tuning of the FPI. The initial value of the magnetic field is B_0. The system's ground state is described by the charge $Q_{\downarrow} = eN_{\downarrow}$ with $N_{\downarrow}$ electrons occupying the LLL, and the charge $Q_{\uparrow} = eN_{\uparrow}$ with $N_{\uparrow}$ electrons located at the compressible bulk puddle. The edge of the FPI has the initial boundary of the incompressible $\nu = 1$ area $\bar{A}$, dictated by the chemical potential of the leads. We will argue below that this boundary may be modified by varying the magnetic field B and the modulation gate voltage V_{MG}. In order to determine the ground state configuration $(N_{\downarrow}, N_{\uparrow})$ for the (B, V_{MG}) plane, we first calculate the total electrostatic energy.

We note that even if the number of electron occupying the $\nu = 1$ level in the FPI remains unchanged (i.e. no transfer of electrons to/from the bulk puddle or the leads), charging of the edge can be induced by varying the magnetic field $\delta B = B - B_0$. The excess charge on the edge is then:

$$\delta q_3 - \delta q_1 = \delta Q_{\downarrow} - e \cdot \frac{A\delta B}{\phi_0} = e\delta N_{\downarrow} - e \cdot \frac{A\delta B}{\phi_0}. \qquad (1)$$

The compressible puddle at the center of the FPI, serves as an effective reservoir: it may take out or give away charge from/to the edge, then minimizing the electrostatic energy. The excess charge in the bulk is expressed as:

$$-\delta q_2 - \delta q_3 = \delta Q_{\uparrow} + e \cdot \frac{A\delta B}{\phi_0} = e\delta N_{\uparrow} + e \cdot \frac{A\delta B}{\phi_0}. \qquad (2)$$

For a fixed $\delta N_{\uparrow}$, the variation $\delta B > 0$ leads to charge accumulation in the bulk of the $\nu = 1$ Landau level by $e \cdot \frac{A\delta B}{\phi_0}$. As a matter of fact, the charge in the bulk of the FPI is the sum of two contributions: the electrons of which the incompressible $\nu = 1$ liquid is comprised of, and the electrons forming the compressible puddle in the middle. The total electrostatic energy of the electric circuit is expressed as:

$$E_{\mathrm{total}} = \frac{q_1^2}{2C_{\mathrm{edge}}} + \frac{q_2^2}{2C_{\mathrm{bulk}}} + \frac{q_3^2}{2C_{\mathrm{eb}}} - q_1\delta V_{\mathrm{MG}} - q_2\delta V_{\mathrm{MG}}$$

$$= \frac{K_{\mathrm{E}}}{2}e^2 \cdot \left(\delta N_{\downarrow} - \frac{A\delta B}{\phi_0} - \frac{\alpha B_0 \delta V_{\mathrm{MG}}}{\phi_0}\right)^2 + \frac{K_{\mathrm{EB}}}{2}e^2 \cdot \left(\delta N_{\downarrow} + \delta N_{\uparrow} - \frac{\gamma \delta V_{\mathrm{MG}}}{|e|}\right)^2 + \frac{K_{\mathrm{B}}}{2}e^2$$

$$\cdot \left(\delta N_{\uparrow} + \frac{A\delta B}{\phi_0} + \frac{\alpha B_0 \delta V_{\mathrm{MG}}}{\phi_0} - \frac{\gamma \delta V_{\mathrm{MG}}}{|e|}\right)^2 - \frac{C_{\mathrm{edge}} + C_{\mathrm{bulk}}}{2}(\delta V_{\mathrm{MG}})^2. \qquad (3)$$

Here, $\mathrm{K_E} = \frac{\mathrm{C_{bulk}}}{\mathrm{D}}, \mathrm{K_B} = \frac{\mathrm{C_{edge}}}{\mathrm{D}}$, $\mathrm{K_{EB}} = \frac{C_{\mathrm{eb}}}{\mathrm{D}}$, and $\mathrm{D} = (\mathrm{C_{eb}} + \mathrm{C_{edge}})(\mathrm{C_{eb}} + \mathrm{C_{bulk}}) - \mathrm{C_{eb}^2}$. In terms of the AB phase Supplementary Equation 3 reads:

$$E_{\mathrm{total}} = \frac{K_{\mathrm{E}}}{2}e^2 \cdot \left(\delta N_{\downarrow} - \frac{\delta\phi_{\mathrm{AB}}}{\phi_0}\right)^2 + \frac{K_{\mathrm{EB}}}{2}e^2 \cdot \left(\delta N_{\downarrow} + \delta N_{\uparrow} - \frac{\gamma \delta V_{\mathrm{MG}}}{|e|}\right)^2 + \frac{K_{\mathrm{B}}}{2}e^2$$

$$\cdot \left(\delta N_{\uparrow} - \left(\frac{\gamma \delta V_{\mathrm{MG}}}{|e|} - \frac{\delta\phi_{\mathrm{AB}}}{\phi_0}\right)\right)^2 - \frac{C_{\mathrm{edge}} + C_{\mathrm{bulk}}}{2}(\delta V_{\mathrm{MG}})^2. \qquad (4)$$

We will disregard the last two terms since they do not depend on the number of the electrons, and hence play no role in determining the ground state; evidently, dropping them results in Eq. 4 in the main text. The parameter $\alpha = \frac{C_{\mathrm{edge}}}{B_0|e|}\phi_0$ effectively accounts for changes of the area by MG voltage variations δV_{MG}, while keeping $\delta Q_{\downarrow}$ unchanged, and the parameter γ is defined as $C_{\mathrm{edge}} + C_{\mathrm{bulk}}$. We emphasize that the presence of an Ohmic-contact renormalizes the values of capacitances C_{edge} and C_{bulk} upwards, while the value of C_{eb} is kept unchanged, thus reducing the charging energy. When we additionally account for the effect of the Ohmic contact through the mutual capacitance, C_{bo} (C_{eo}), between the bulk (or the edge) and the Ohmic contact, C_{bulk} renormalizes to $C_{\mathrm{bulk}} + C_{\mathrm{bo}}$ (this is due to the parallel connection of C_{bulk} with C_{bo}). The effective value of $\frac{K_{\mathrm{EB}}}{K_{\mathrm{E}}} = \frac{C_{\mathrm{eb}}}{C_{\mathrm{bulk}}}$ is then scaled down, pushing the regime of behavior of the FPI more towards the AB regime.

Relation to previous works

In the work by Halperin *et al.*[1] the energy of the system was formulated as follows:

$$E = \frac{K_{\mathrm{I}}}{2}(\delta n_{\mathrm{I}})^2 + \frac{K_{\mathrm{L}}}{2}(\delta n_{\mathrm{L}})^2 + K_{\mathrm{IL}}\delta n_{\mathrm{I}}\delta n_{\mathrm{L}}. \qquad (5)$$

The first two terms in this expression are physically (up to the coefficients) equivalent to the first and third terms in Supplementary Equation 3 & 4; namely:

$$\delta n_{\mathrm{I}} = \delta Q_{\downarrow} - e \cdot \frac{\delta\phi_{\mathrm{AB}}}{\phi_0} = e \cdot \frac{B\delta A_{\mathrm{res}}}{\phi_0}. \qquad (6a)$$

$$\delta n_{\mathrm{L}} = \delta Q_{\uparrow} + e \cdot \frac{\delta \phi_{\mathrm{AB}}}{\phi_0} - \gamma \delta V_{\mathrm{MG}}. \qquad (6b)$$

$$\delta Q_{\mathrm{tot}} = \delta n_{\mathrm{I}} + \delta n_{\mathrm{L}} + \gamma \delta V_{\mathrm{MG}}. \qquad (6c)$$

Now physically the interpretation of the third term in the energy above differs from that of the first term in our energy; $K_{\mathrm{IL}} \cdot \delta n_{\mathrm{I}} \delta n_{\mathrm{L}}$ represents an effective interaction between the edge and the bulk, while $\frac{K_{\mathrm{EB}}}{2} (\delta Q_{\mathrm{tot}})^2$ represents an effective total charging energy. The relation between the different coefficients reads:

$$K_{\mathrm{IL}} = K_{\mathrm{EB}}. \qquad (7a)$$

$$K_{\mathrm{I}} = K_{\mathrm{EB}} + K_{\mathrm{E}}. \quad (7b)$$

$$K_{\mathrm{L}} = K_{\mathrm{EB}} + K_{\mathrm{B}}. \quad (7c)$$

And we can identify at ease that $\Delta = \xi$, where $\Delta \in [0,1]$ is the parameter that denotes the regime in Halperin *et al.*'s model[1].

Calculation of the conductance

In this section, we derive an expression of the conductance through a FPI. For a fixed $\delta N_{\uparrow}$, the total transmission through the FPI is expressed as $T_{\delta N_{\uparrow}} = \left|\tau_{\delta N_{\uparrow}}\right|^2$ with the total transmission amplitude $\tau_{\delta N_{\uparrow}} = t_{\mathrm{l}} t_{\mathrm{r}} \left(1 + \sum_{\mathrm{n}=1}^{\infty} (r_{\mathrm{l}} r_{\mathrm{r}})^n e^{2\pi \cdot i \cdot n \delta \phi_{\mathrm{tot}}/\phi_0}\right)$, considering the summation over all possible number of roundtrips n of an interfering electron. Here r_{l} (r_{r}) and t_{l} (t_{r}) are the reflection and transmission amplitudes of the left (right) QPC, respectively. This total transmission is simplified as

$$T_{\delta N_{\mathrm{bulk}}} = \frac{T_{\mathrm{L}} T_R}{1 + R_{\mathrm{L}} R_{\mathrm{R}} - 2\sqrt{R_{\mathrm{L}} R_{\mathrm{R}}} \cos(2\pi \delta \phi_{\mathrm{tot}}/\phi_0)}, \qquad (8)$$

where $T_{\mathrm{L,R}} = 1 - R_{\mathrm{L,R}} = \left|t_{\mathrm{l,r}}\right|^2 = 1 - \left|r_{\mathrm{l,r}}\right|^2$. In the single particle picture, $\delta \phi_{\mathrm{tot}}$ is determined by the ratio of the highest energy level ($\epsilon = -\frac{\delta \phi_{\mathrm{AB}}}{\phi_0} \Delta + K_{\mathrm{EB}} \left(e \cdot \delta N_{\uparrow} + e \cdot \frac{\delta \phi_{\mathrm{AB}}}{\phi_0} - \gamma \delta V_{\mathrm{MG}}\right)$) below Fermi energy to the level spacing ($\Delta = K_{\mathrm{E}} + K_{\mathrm{EB}}$),

$$\frac{\delta \phi_{\mathrm{tot}}}{\phi_0} \equiv -\frac{\epsilon}{\Delta} = \frac{\delta \phi_{\mathrm{AB}}}{\phi_0} - \xi \left(\delta N_{\uparrow} + \frac{\delta \phi_{\mathrm{AB}}}{\phi_0} - \frac{\gamma \delta V_{\mathrm{MG}}}{|e|}\right). \qquad (9)$$

Two comments are due here: Supplementary Equation 9 coincides with a single-particle analysis of transmission through the FPI. There are two facets of many-body physics that are neglected here. (i) Putting aside higher order processes in the tunneling amplitudes $\{\Gamma\}$, we can neglect inelastic transmission processes that leave a trace on the FPI (By contrast, cf. Ref. 5, there are scenarios where

inelastic contributions are of the same order as elastic ones). (ii) Assuming $K_{\mathrm{EB}} \ll K_{\mathrm{B}}$, we may ignore the back action of varying the number of electrons in the compressible puddle due to a change of the edge configuration.

When $\delta N_{\uparrow}$ fluctuates, Supplementary Equation 8 should be modified as $\langle T\rangle = \sum_{\delta N_{\uparrow}} P_{\delta N_{\uparrow}} T_{\delta N_{\uparrow}}$, where $P_{\delta N_{\uparrow}}$ is the probability that the number of the electrons in the compressible puddle is $\delta N_{\uparrow}$. In order to calculate $P_{\delta N_{\uparrow}}$, we first start with the occupation number $\langle \delta N_{\uparrow}\rangle$. Within the additional approximation of $\Gamma_{\mathrm{bulk}} \ll (K_{\mathrm{B}} + K_{\mathrm{EB}})$, while keeping $\Gamma_{\mathrm{bulk}} > 0$, the system of the compressible puddle can be treated as a two-state system, an occupied (o) and unoccupied (u) states; this terminology refers to the closest level to the Fermi level; fluctuation of $\delta N_{\uparrow}$ that go further than ± 1 are neglected. The state of the compressible puddle is then expressed as $\sqrt{P_u}|u\rangle + \sqrt{P_u}|o\rangle$, where P_o (P_u) is the probability for an (un)occupied state of the said level with $P_o = \langle \delta N_{\uparrow}\rangle - F[\langle \delta N_{\uparrow}\rangle]$ $(P_u = 1 - P_o)$ and $F[x]$ is the integer part of x. The occupation number $\langle \delta N_{\uparrow}\rangle$ is determined by the integral of the single particle bulk states centered at $\epsilon_n = (K_{\mathrm{B}} + K_{\mathrm{EB}})(n + \frac{\delta\phi_{\mathrm{AB}}}{\phi_0} - \frac{\gamma\delta V_{\mathrm{MG}}}{|e|})$ over the energy as:

$$\langle \delta N_{\uparrow}\rangle \equiv \sum_{n=0}^{\infty} \frac{1}{\pi}\int_{-\infty}^{0} \frac{\Gamma_{\mathrm{bulk}}}{(\epsilon - \epsilon_n)^2 + \Gamma_{\mathrm{bulk}}^2} d\epsilon$$

$$= \sum_{n=0}^{\infty} \left(\frac{1}{2} - \tan^{-1}\left[\frac{K_{\mathrm{B}} + K_{\mathrm{EB}}}{\Gamma_{\mathrm{bulk}}}\left(n + \frac{\delta\phi_{\mathrm{AB}}}{\phi_0} - \frac{\gamma\delta V_{\mathrm{MG}}}{|e|}\right)\right]\right). \quad (10)$$

This conductance is related with $\langle T\rangle$, which is the average over the different possible values of $\delta N_{\uparrow}$ with the corresponding probabilities:

$$G = \frac{e^2}{h}\langle T\rangle = \frac{e^2}{h}\left(P_u T_{F[\langle \delta N_{\uparrow}\rangle]} + P_o T_{F[\langle \delta N_{\uparrow}\rangle]+1}\right). \quad (11)$$

This equation is used for drawing the Figure 4b and Supplemental Figures 8c and 9b.

Charge stability diagram

In order to formally express the charge stability diagram we first optimize the energy in Supplementary Equation 4 with respect to both δA_{res} and $\delta Q_{\uparrow}$ at the same, resulting in:

$$B\delta A_{\mathrm{res}} = 0. \quad (12a)$$

$$\delta Q_{\uparrow} = -e \cdot \frac{A\delta B}{\phi_0} - \left(e \cdot \frac{\alpha B}{\phi_0} - \gamma\right)\delta V_{\mathrm{MG}}. \quad (12b)$$

Every time these two equations are satisfied, all regimes coincide since both the dipole and the charging energies are at local minima (in fact, it is clear that plugging Supplementary Equation 12 in Supplementary Equation 4 results in $\delta E = 0$). The charge-states which satisfy Supplementary Equation 10, $(\delta N_{\downarrow}, \delta N_{\uparrow}) = (n, m)$ produce the vectors:

$$(\delta B, \delta V_{\mathrm{MG}}) = \left(\Delta B^{(\mathrm{AB})} \left(n - \frac{\Delta V_{\mathrm{MG}}^{(\mathrm{CD})}}{\Delta V_{\mathrm{MG}}^{(\mathrm{AB})}} \cdot (n+m) \right), \quad \Delta V_{\mathrm{MG}}^{(\mathrm{CD})} \cdot (n+m) \right), \quad (13)$$

which describe the vectors of the charge-stability diagram. Similar to a Bravais lattice, these vectors may be spanned by two:

$$\mathbf{a}_1 = \left(0, \Delta B^{(\mathrm{AB})}\right), \qquad (14a)$$

$$\mathbf{a}_2 = \left(\Delta V_{\mathrm{MG}}^{(\mathrm{CD})}, -\Delta \mathrm{B}^{(\mathrm{AB})} \frac{\Delta V_{\mathrm{MG}}^{(\mathrm{CD})}}{\Delta V_{\mathrm{MG}}^{(\mathrm{AB})}} \right), \qquad (14b)$$

so that the lattice is given by $\mathbf{R}_{n,m} = \sum_{n,m} n \cdot \mathbf{a}_1 + m \cdot \mathbf{a}_2$. Hence the reciprocal lattice is given by the four equations $\mathbf{a}_j \cdot \mathbf{b}_i = 2\pi \delta_{ij}$ which results in the:

$$\mathbf{b}_1 = 2\pi \cdot \omega_{\mathrm{AB}} = \left(\frac{2\pi}{\Delta V_{\mathrm{MG}}^{(\mathrm{CD})}}, \frac{2\pi}{\Delta B^{(\mathrm{AB})}} \right). \quad (15a)$$

$$\mathbf{b}_2 = 2\pi \cdot \omega_{CD} = \left(\frac{2\pi}{\Delta V_{\mathrm{MG}}^{(\mathrm{CD})}}, 0 \right). \qquad (15b)$$

These are indeed the underlying AB and CD frequencies, as anticipated.

Now it is clear from Supplementary Equation 14 above that, the vectors spanning the charge stability diagram do not depend on the regime. Nevertheless, the different regimes' charge stability diagram (CSD) differs by the shape of the unit-cells, as we show in the different asymptotic cases in the following section. The full CSD including the structures of the unit-cells is obtained by finding $(\delta N_{\downarrow}, \delta N_{\uparrow})$ which minimize the energy (Supplementary Equation 4). In this way, Supplementary Figures 7 & 8a, discussed in what follows, are simulated.

The Aharonov-Bohm and Coulomb-dominated regime

First we discuss the two distinct regimes of behavior of the FPI: the Aharonov-Bohm (AB) regime and the Coulomb-dominated (CD) regime[1,4]. These are distinguished by the slope of their equi-phase

lines in the 2D conductance as function of magnetic field δB and modulation gate voltage δV_{MG}(the so-called pajama patterns). In the AB regime, the equi-phase lines have a negative slope; when the magnetic field increases, the phase remains invariant as the modulation gate voltage (and thus the area) decreases. On the other hand, in the CD regime, equi-phase lines follow a non-negative slope. The different regimes can be retrieved by examining the asymptotic limits of the energy given in Supplementary Equation 3; we shall discuss, for each of the asymptotic limits, its physical interpretation and its charge stability diagram.

AB regime: Two asymptotic choices of parameters yield this regime, discussed separately below. In both cases, $K_{\mathrm{E}} \gg K_{\mathrm{EB}}$, namely $\xi = 0$.

(i) A truly non-interacting system, expressed in terms of the effective capacitances: $C_{\mathrm{edge}} \to \infty, C_{\mathrm{bulk}} \to \infty, C_{\mathrm{eb}} \to \infty$ (see Supplementary Figure 6a). In this regime, $K_{\mathrm{B}} = 0, K_{\mathrm{EB}} = 0$, and K_{E} corresponds to the level spacing of the single-particle energies, a scale which is not included in Supplementary Equation 3. K_{E} is then determined by the slope of the confining potential at the Fermi level, as well as the magnetic field B, and the area $\bar{A}$. Since $K_{\mathrm{B}} = 0$, the charge imbalance in the bulk does not play a role in determining the ground state configuration so that the CSD describes the charge state of the edge solely.

(ii) More interestingly, and experimentally relevant, it is possible to obtain a negative slope of the constant phase lines for an interacting system (see Supplementary Figure 6b) by requiring $C_{\mathrm{bulk}} \gg C_{\mathrm{eb}}$ and $K_{\mathrm{E}} \gg K_{\mathrm{EB}}$. This result is equivalent to our measurements with the AB-dominated device shown in Fig. 2a in the main text.

CD regime: Here too, we consider two asymptotic scenarios.

(iii) $C_{\mathrm{bulk}} \ll C_{\mathrm{eb}}$ and $C_{\mathrm{edge}} > C_{\mathrm{bulk}}$ ($K_{\mathrm{E}} \ll K_{\mathrm{EB}}$, $K_{\mathrm{E}} < K_{\mathrm{B}}$) (see Supplementary Figure 6c). In this regime, the lines of constant phase and the lines of conductance maxima are vertical in the $(\delta B, \delta V_{\mathrm{MG}})$ plane. If one crosses the blue lines seen in Supplementary Figure 6c, redistribution of the electrons between the bulk and the edge takes place. This result is equivalent to our measurements with the CD device shown in Fig. 2b in the main text.

(iv) $C_{\mathrm{bulk}}, C_{\mathrm{edge}} \ll C_{\mathrm{eb}}$ ($K_{\mathrm{E}}, K_{\mathrm{B}} \ll K_{\mathrm{EB}}$) (cf. Supplementary Figure 6d). While the lines of constant phase and the lines of conductance maxima are vertical in the scenario (i) of the CD regime (cf. Supplementary Figure 6c), here vertical boundaries consist of alternating black lines (where $\delta N_{\uparrow}$ is changed by ± 1 with constant $\delta N_{\downarrow}$) and the red lines (where $\delta N_{\downarrow}$ is changed by ± 1 with constant $\delta N_{\uparrow}$).

The intermediate regime

Finally, we discuss the intermediate regime[6] between the asymptotic AB and CD regimes. As shown in Supplementary Figure 7a, the conductance peak (red) lines with a negative slope (the signature of the AB regime) separate states with different total number of electrons, $\delta N_{\uparrow} + \delta N_{\downarrow}$ (the signature of the CD regime). The intermediate regime can be described by the parameters $0 < \frac{K_{\mathrm{EB}}}{K_{\mathrm{E}}+K_{\mathrm{EB}}} < 1$, as compared with $\frac{K_{\mathrm{EB}}}{K_{\mathrm{E}}} = 0$ for the AB regime and $\frac{K_{\mathrm{EB}}}{K_{\mathrm{E}}+K_{\mathrm{EB}}} = 1$ for the CD regime. The conductance in the $(\delta B, \delta V_{\mathrm{MG}})$ plane based on the capacitive model is plotted in Supplementary Figure 7c (low transmission) and Supplementary Figure 8b (high transmission), and they are compared to the experimental results (Supplementary Figure 7b and 8a, respectively).

Supplementary Note 3. High transmission approximation

Conductance

In the high transmission limit (namely, $T \lesssim 1$), and assuming $\Gamma_{\mathrm{bulk}} = 0$, we may approximate:

$$G(T \lesssim 1) = \frac{e^2}{h} T^2 (1 + R^2 \cos(2\pi \cdot \delta\phi_{\mathrm{tot}}/\phi_0)). \qquad (16)$$

This expression is used for the theoretical graphs in Fig. 5 in the main text.

Most generally, for any setting of our two external knobs δB and δV_{MG}, the system optimizes its energy by setting δA_{res} & $\delta Q_{\uparrow}$ (or equivalently $\delta Q_{\downarrow}$ & $\delta Q_{\uparrow}$), according to the energy given in Supplementary Equation 4. The high transmission limit is translated into relaxing the assumption of quantization of $\delta Q_{\downarrow}$, which is made in Supplementary Note 2. In this way we can retrieve the total phase in a straightforward fashion by optimizing the energy with respect to δA_{res}, for a given value of $\delta Q_{\uparrow} \in [0, \pm 1, \pm 2, \dots]$:

$$\frac{\partial \delta E}{\partial \delta A_{\mathrm{res}}} = K_{\mathrm{EB}} \cdot (\delta Q_{\mathrm{tot}} - \gamma \delta V_{\mathrm{MG}}) \cdot \frac{B}{\phi_0} + K_{\mathrm{E}} \cdot \left(\delta Q_{\downarrow} - e \cdot \frac{\delta\phi_{\mathrm{AB}}}{\phi_0}\right) \cdot \frac{B}{\phi_0}, \qquad (17)$$

setting now $\frac{\partial \delta E}{\partial \delta A_{\mathrm{res}}} = 0$ we get:

$$e \cdot \frac{B \delta A_{\mathrm{res}}}{\phi_0} = \xi \left(\gamma \delta V_{\mathrm{MG}} - e \cdot \frac{\delta\phi_{\mathrm{AB}}}{\phi_0}\right) - \xi \cdot \delta Q_{\uparrow}\,. \qquad (18)$$

Now, once plugged into the total phase we get:

$$\frac{\delta\phi_{\mathrm{tot}}}{\phi_0} = \frac{\delta\phi_{\mathrm{AB}}}{\phi_0} + \xi \left(\frac{\gamma \delta V_{\mathrm{MG}}}{e} - \frac{\delta\phi_{\mathrm{AB}}}{\phi_0}\right) - \xi \cdot \frac{\delta Q_{\uparrow}}{e}\,, \qquad (19a)$$

or:

$$\frac{\delta\phi_{\text{tot}}}{\phi_0} = (1-\xi)\cdot\frac{\delta\phi_{\text{AB}}}{\phi_0} + \xi\cdot\frac{\gamma\delta V_{\text{MG}}}{e} - \xi\cdot\frac{\delta Q_\uparrow}{e}\ . \qquad (19b)$$

Now the first two terms are continuously-varying variables which describe the interference phase of electrons in the lowest Landau level for a constant $\delta Q_\uparrow$ in the compressible puddle; while the third term describes the phase-jump which occurs once $\delta Q_\uparrow$ changes by $\pm e$ (as further explained below). We stress here that the transmission T does not affect the value of ξ, but only the shape of the unit-cells in the CSD as well as the clarity of its lattice-like, via SP states broadening, as shown in Supplementary Figure 5 with both experimental data and theoretical.

Phase-jump lines

As seen in Supplementary Figure 6a, the lines along which $\delta Q_\uparrow$ increments by $\pm e$ are sets of broken lines ('zig-zags'). These zig-zags consist of two types of segments; the first (dark-blue) describes a processes of varying the number of electrons in the compressible puddle $(\delta Q_\uparrow, \delta Q_\downarrow) \to (\delta Q_\uparrow \pm e, \delta Q_\downarrow)$; and the second (light-blue) describes 'reshuffling' of the charge between the edge and the bulk $(\delta Q_\uparrow, \delta Q_\downarrow) \to (\delta Q_\uparrow \pm e, \delta Q_\downarrow \mp e)$.

Nonetheless, for higher transmission probabilities, these zig-zags become straight lines. These results can be easily obtained be rewriting the energy in the following form:

$$E_{\text{total}} = \frac{\text{K}_{\text{E}}}{2}\left(\delta Q_\downarrow - e\cdot\frac{\delta\phi_{\text{AB}}}{\phi_0} + \frac{K_{\text{EB}}}{K_{\text{E}}}\cdot\left(\delta Q_\uparrow - \left(\gamma\delta V_{\text{MG}} - e\cdot\frac{\delta\phi_{\text{AB}}}{\phi_0}\right)\right)\right)^2 + \frac{\left(K_{\text{E}} + \frac{K_{\text{EB}}^2}{K_{\text{E}}}\right)}{2}\left(\delta Q_\uparrow - \left(\gamma\delta V_{\text{MG}} - e\cdot\frac{\delta\phi_{\text{AB}}}{\phi_0}\right)\right)^2 . \quad (20)$$

Since at the high transmission limit we consider $\delta Q_\downarrow$ to be a continuous variable, it is evident that the first term in this energy is zero at all times (since $\delta Q_\downarrow$ appears only within it. Thus the energy effectively depends on the second term only, which is clearly minimized by setting $\delta Q_\uparrow = \left(\gamma\delta V_{\text{MG}} - e\cdot\frac{\delta\phi_{\text{AB}}}{\phi_0}\right)$. Writing this expression for any $\delta Q_\uparrow = ne$ with n an integer results in the line in the $B - V_{\text{MG}}$ plane along which having exactly $\delta Q_\uparrow = ne$ is optimal. Thus, very intuitively, the lines along which $\delta Q_\uparrow$ changes by ± 1 may be obtained by setting $\delta Q_\uparrow = (n+\frac{1}{2})\cdot e$:

$$\frac{A}{\phi_0}\delta B + \left(\frac{\alpha B}{\phi_0} - \frac{\gamma}{e}\right)\delta V_{\text{MG}} = n + \frac{1}{2}\ . \quad (21a)$$

or:

$$\frac{\gamma\delta V_{\mathrm{MG}}}{e}-\frac{\delta\phi_{\mathrm{AB}}}{\phi_0}=n+\frac{1}{2}\ . \qquad (21b)$$

By taking a variation on this equation resulting in $\Delta n = 1$ we obtain the PJ lines 2D frequencies (assuming high QPCs transmission):

$$1/\Delta B_{\mathrm{PJ}} = A/\phi_0\ . \qquad (22a)$$

$$\frac{1}{\Delta V_{\mathrm{PJ}}}=\frac{\alpha B}{\phi_0}-\frac{\gamma}{e}\ . \quad (22b)$$

or, in terms of the underlying AB and CD frequencies:

$$1/\Delta B^{(\mathrm{PJ})} = 1/\Delta B^{(\mathrm{AB})}\ . \qquad (23a)$$

$$1/\Delta V_{MG}^{(\mathrm{PJ})} = 1/\Delta V_{MG}^{(\mathrm{AB})} - 1/\Delta V_{MG}^{(\mathrm{CD})}\ . \quad (23b)$$

Supplementary References

1. Halperin, B. I., Stern, A., Neder, I. & Rosenow, B. Theory of the Fabry-Pérot quantum Hall interferometer. *Phys. Rev. B* **83,** 155440 (2011).

2. Chklovskii, D. B., Shklovskii, B. I. & Glazman, L. I. Electrostatics of edge channels. *Phys. Rev. B* **46,** 4026–4034 (1992).

3. Lee, W.-R. & Sim, H.-S. Capacitive interaction model for Aharonov-Bohm effects of a quantum Hall antidot. *Phys. Rev. B* **83,** 035308 (2011).

4. Rosenow, B. & Halperin, B. I. Influence of interactions on flux and back-gate period of quantum hall interferometers. *Phys. Rev. Lett.* **98,** 106801 (2007).

5. König, J. & Gefen, Y. Coherence and partial coherence in interacting electron systems. *Phys. Rev. Lett.* **86,** 3855–3858 (2001).

6. Dinaii, Y., Gefen, Y. & Rosenow, B. Transmission phase lapses through a quantum dot in a strong magnetic field. *Phys. Rev. Lett.* **112,** 246801 (2014).
